# Discovery of a Vertebrate-Specific Factor that Processes Flagellar Glycolytic Enolase during Motile Ciliogenesis

**DOI:** 10.1016/j.isci.2020.100992

**Published:** 2020-03-19

**Authors:** Keishi Narita, Hiroaki Nagatomo, Hiroko Kozuka-Hata, Masaaki Oyama, Sen Takeda

**Affiliations:** 1 Department of Anatomy and Cell Biology, Faculty of Medicine, University of Yamanashi, Chuo, Yamanashi 409-3898, Japan; 2 Center for Life Science Research, University of Yamanashi, Chuo, Yamanashi 409-3898, Japan; 3 Medical Proteomics Laboratory, Institute of Medical Science, The University of Tokyo, 4-6-1 Shirokanedai, Minato-ku, Tokyo 108-8639, Japan

**Keywords:** Rodent Genetics, Rodent Reproduction, Developmental Genetics

## Abstract

Motile cilia and flagella require ATP for their formation and function. Although glycolytic enzymes are components of flagellar proteomes, how they translocate to flagella is unknown. Here we show that the expression pattern of the functionally nonannotated gene *4833427G06Rik* (*C11orf88*), which is found only in vertebrates and is designated here as Hoatzin (*Hoatz*), suggests a functional association of its product with motile cilia and flagella. *Hoatz* knockout (KO) mice developed hydrocephalus and male infertility in an autosomal recessive manner, and the ependymal cilia frequently showed disorganized axonemes, reducing motility associated with collapsed spermatid flagella during cytodifferentiation. HOATZ was associated with certain proteins, including the flagellar glycolytic enzyme ENO4. In the testes of the *Hoatz* KO mice, the immature form of ENO4 accumulated in abnormal cytoplasmic puncta of developing spermatids. These data indicate that HOATZ is required for motile ciliogenesis and flagellar genesis in vertebrates by mediating the maturation of ENO4.

## Highlights

• Knockout of *Hoatz* causes hydrocephalus and oligo-astheno-terato-zoospermia
• Motile cilia are variably affected by the *Hoatz* mutation depending on tissue type
• Candidate HOATZ-interacting proteins including ENO4 are identified
• Knockout of *Hoatz* alters the western blot profile of ENO4

## Graphical Abstract

**Figure:**
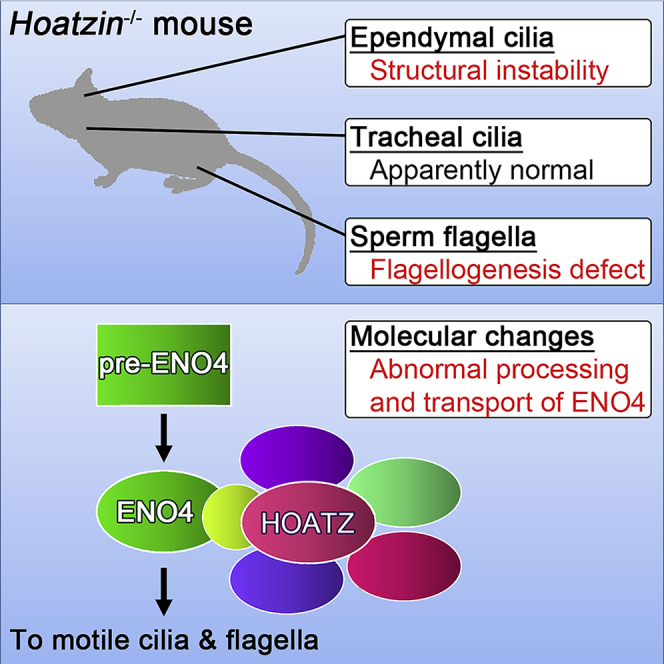

## Introduction

Motile cilia and flagella are subcellular organelles of eukaryotes that propel extracellular fluids. A small number of vertebrate cell types express these structures under the regulation of specific transcription factors (Choksi et al., 2014, Gerdes et al., 2009) that are required for clearing airways, transport of gametes, circulating cerebrospinal fluid, or determining left-right asymmetry (Hirokawa et al., 2006, Zariwala et al., 2006). The typical internal architectures of the 9 + 2 axoneme (Lin et al., 2015); biogenesis, including the cytoplasmic preassembly of dynein arms (Olcese et al., 2017, Omran et al., 2008); and subsequent intraflagellar transport (Snell et al., 2004) are well conserved among eukaryotic species. ATP is required for their formation and motility through ATP-driven motor proteins such as kinesins and dyneins (Khan and Scholey, 2018). The flagellar proteome of *Chlamydomonas reinhardtii* comprises glycolytic enzymes, indicating the importance of *in situ* ATP production for flagellar motility (Mitchell, 2005, Pazour et al., 2005). Such glycolytic enzymes are involved in the second half of the glycolysis pathway (pay-off phase) in which the high-energy glucose metabolite glyceraldehyde 3-phosphate is converted into low-energy products (e.g., pyruvate) to produce ATP (Berg et al., 2011). However, insufficient data are available to show that these metabolic enzymes are actively transported into motile subcellular organelles or if their molecular mechanisms are conserved among eukaryotes.

Here we characterized a mouse gene of unknown function designated *4833427G06Rik* (*C11orf88*), which is identified in a group of 99 putative cilia-related genes in mammals (McClintock et al., 2007). Our unpublished analysis of *4833427G06Rik* revealed that its sequence is conserved only in vertebrates. We designate this gene here as Hoatzin [*Hoatz*] according to the **h**ydrocephalus and **o**ligo-**a**stheno-**t**erato-**z**oospermia phenotype of *Hoatz* KO mice. We therefore hypothesized that its product (HOATZ) contributes to a vertebrate-specific function associated with motile cilia and flagella. Moreover, our present data suggest that HOATZ mediates motile ciliogenesis by processing ENO4, a glycolytic enzyme expressed specifically in ciliated cells of vertebrates.

## Results

### Hoatzin mRNA Is Specifically Expressed in Tissues with Motile Cilia and Flagella

**Figure 1:**
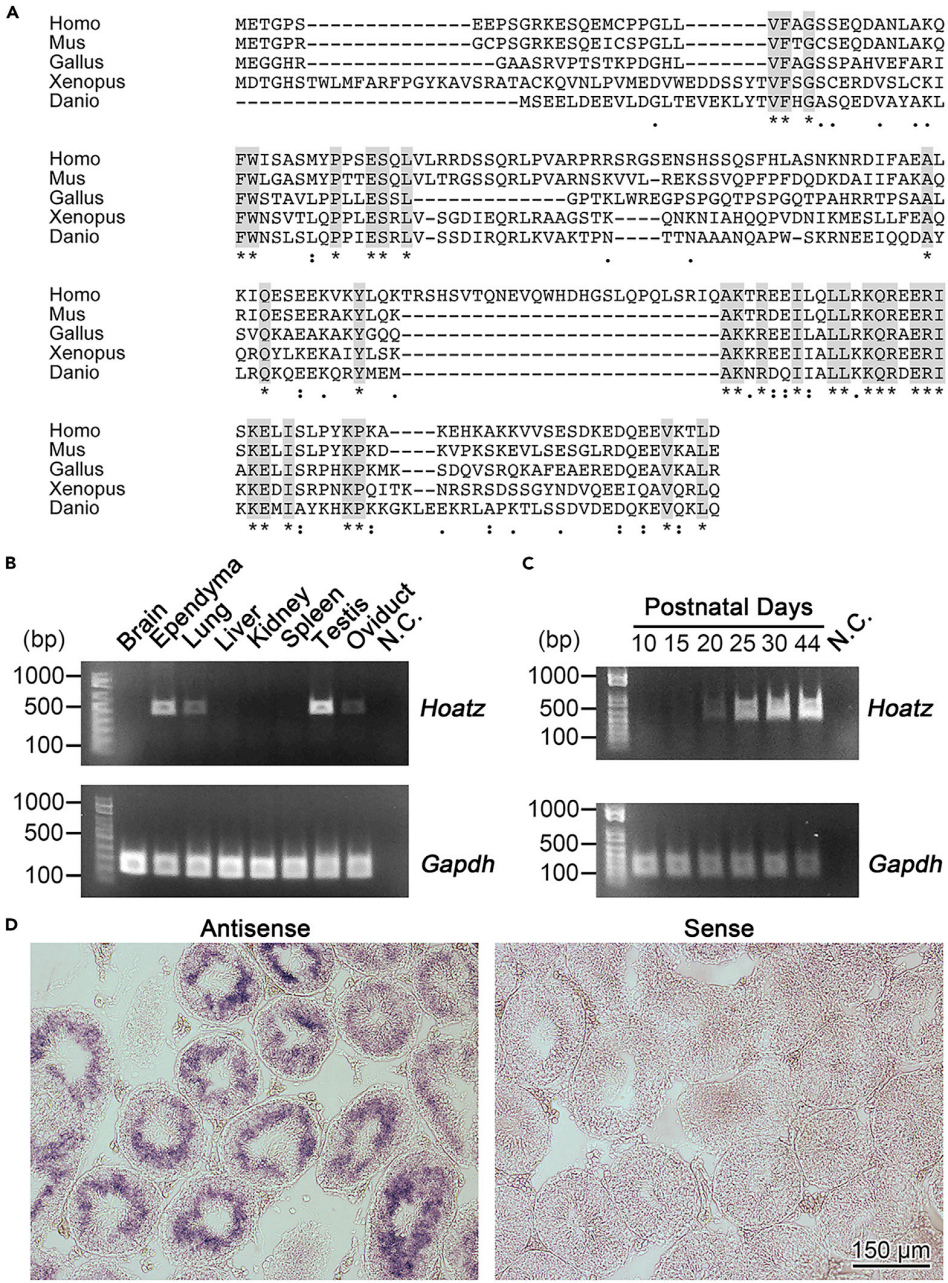
*Hoatzin* mRNA Is Specifically Expressed in Tissues with Motile Cilia and Flagella (A) The amino acid sequence alignment of HOATZ with those of proteins of diverse species was generated using the MUSCLE program (Edgar, 2004). (B) Semiquantitative reverse-transcriptase polymerase chain reaction (RT-PCR) analysis of *Hoatz* expression using RNAs extracted from the mouse brain, ependyma (primary culture), lung, liver, kidney, spleen, testis, and oviduct. N.C., negative control (minus template). *Gapdh* mRNA served as a control. (C) Changes in mRNA expression levels of *Hoatz* during the first wave of spermatogenesis. N.C., negative control (minus template). *Gapdh* mRNA served as a control. (D) Detection of *Hoatz* mRNA in mouse testis using RNA *in situ* hybridization. The tissue sections were incubated with antisense and sense probes. The absence of a signal in the section incubated with the sense probe indicates the specificity of the hybridization. See also Figure S1 and Video S1.

During our search for novel cilia-related genes in mice, we identified a functionally nonannotated gene designated *4833427G06Rik*, which we named Hoatzin (*Hoatz*) according to the knockout (KO) phenotype described above and encodes a 19 kDa (147 amino acid residues) protein without a known functional domain. Significant amino acid sequence similarities were detected only among vertebrate proteins, including a highly conserved region near the C terminus (Figure 1A). Reverse-transcriptase polymerase chain reaction (RT-PCR) analysis of adult mouse tissues and primary cultured ependyma strongly suggest that gene expression was limited to the cells with motile cilia or flagella (Figure 1B). Furthermore, *Hoatz* mRNA was detected in cultured brain ependyma, lung, testis, and oviduct but not in whole brain, liver, kidney, spleen, and eyeball (Figure 1B and data not shown). *Hoatz* expression in testes was detected as early as postnatal day 15 (P15), which then continually increased during the first 45 days (Figure 1C). The first wave of spermatogenesis occurs during this time when primitive spermatogonia in the testes synchronously differentiate, and new populations of germ cells consecutively appear as follows: spermatocytes, P15; spermatids, P21; and spermatozoa, P42 (Zimmermann et al., 2015). RNA *in situ* hybridization analysis of a 4-week-old mouse testis detected *Hoatz* expression predominantly in haploid spermatids undergoing flagellogenesis at the luminal side of the seminiferous tubules (Figure 1D).

The subcellular localization of HOATZ was investigated using cultured ependyma expressing recombinant HOATZ-FLAG (Figure S1A). Immunostaining of the transduced cells detected the majority of the protein in the cytoplasm, although it was occasionally detected in the cilia (Figure S1B). When a small hairpin RNA (shRNA) targeting *Hoatz* mRNA was expressed in cultured ependyma, one (TRCN0000201969) of five clones exhibited abnormal ciliogenesis (data not shown). These observations suggest the importance of HOATZ for the motile cilia. Moreover, lentiviral transduction of *Hoatz* cDNA into the ependyma of a *Hoatz*^*−/−*^ mouse rescued the phenotype of low ciliary beating frequency (Figure S1C). Immunostaining of the transduced *Hoatz*^*−/−*^ ependyma using an anti-FLAG antibody detected the tagged HOATZ in the cilia (Figure S1D).

### Knockout of Hoatz Causes Hydrocephalus and Oligo-Astheno-Terato-Zoospermia

We mutated *Hoatz* using CRISPR/Cas9 technology (Figure S2A) as described in detail in the Methods section. Among the six founder mutants screened using tail genome sequencing, only one transmitted mutations to the progeny. However, this female founder had chimeric mutations in germline cells and produced three strains, each with different indels (Figures S2B and S2C). Strain #1 had a single deletion of guanine at the double-strand break site, c31, resulting in a frameshift. Strain #2 had the same deletion but with insertion of CTA, again causing a frameshift. Strain #3 had a 114-nucleotide deletion around the target sequence and a 381-nucleotide insertion downstream of the target sequence. Strain #3 was mainly used because of the simplicity of its genotyping (Figure S2D). HOATZ expression in strain #3 was undetectable using a HOATZ-specific rabbit polyclonal antibody (Figures S2E–S2G).

**Figure 2:**
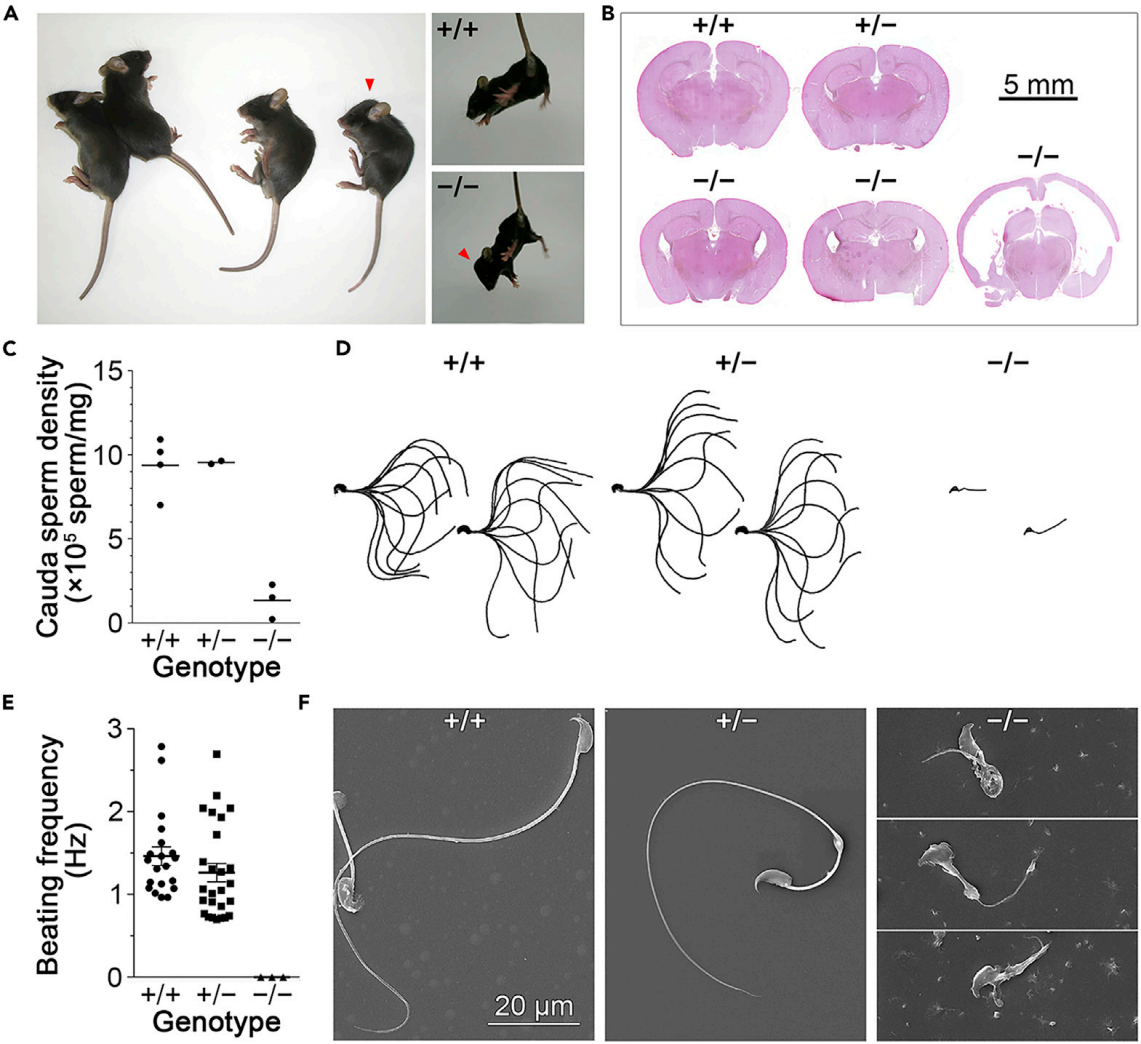
Knockout of *Hoatz* Causes Hydrocephalus and Oligo-Astheno-Terato-Zoospermia (A) Four-weeks-old male littermates of *Hoatz*^*+/−*^ heterozygous parents. One mouse with a *Hoatz*^*−/−*^ genotype exhibited a dome-like skull (arrowhead) characteristic of hydrocephalus. (B) H&E-stained coronal section of the brain from the 4-week-old littermates described above. Note the enlarged lateral ventricles in the three *Hoatz*^*−/−*^ mutants compared with those of the wild-type and heterozygote. (C) Scatterplots with median lines showing the cauda epididymal sperm density of sexually mature littermates. Note that the *Hoatz*^*−/−*^ spermatozoa did not have motile full-length flagella (n = 4, 2, and 3 for wild-type, heterozygote, and null, respectively). (D) High-speed video microscopy of the sperm-flagellar beating forms. We superimposed 10 frames at equal intervals representing one beating cycle. (E) Scatterplots and the mean ± SEM showing the distributions of the sperm flagella beating frequency (n = 20, 26, and 3 for wild-type, heterozygote, and null mutant, respectively). (F) Representative SEM images of the epididymal spermatozoa of sexually mature littermates. See also Figure S2.

*Hoatz*^*−/−*^ mice exhibited hydrocephalus and oligo-astheno-terato-zoospermia (Figure 2), which was inherited in an autosomal recessive manner and observed in the progeny of six generations of backcrosses with a C57BL/6N background. Varying severities of hydrocephalus were observed in the *Hoatz*^*−/−*^ mutants (Figures 2A and 2B). Some mice developed hydrocephalus rapidly and died before weaning (6 of 36), whereas others developed ventriculomegaly without apparent changes in the shape of the skull (4 of 5). In contrast, oligo-astheno-terato-zoospermia was consistently associated with the *Hoatz*^*−/−*^ males (33 of 33), causing infertility. The mean testicular size of the *Hoatz*^*−/−*^ mutants was smaller compared with that of wild-type (139 ± 20.3 mg, n = 5; versus 166 ± 2.6 mg, n = 3; wet weight of one pair per animal on week 7). Sperm was collected from cauda epididymis by a swim-out protocol, revealing the mutant sperm count to be approximately 15% of wild-type values (Figure 2C). The recovery of sperm using this swim-out protocol may have been lower in the *Hoatz*^*−/−*^ mice compared with the wild-type samples because of the lack of flagellar motility. We therefore employed another protocol in which semen in the epididymal tail was pushed out using forceps (Nagy et al., 2003). Although the semen was too viscous to accurately measure using a micropipette, the concentrations were approximately 2 × 10^8^ and 1 × 10^9^ cells/mL for *Hoatz*^*−/−*^ and wild-type mice. The epididymal sperm of the *Hoatz*^*−/−*^ mutants exhibited severe morphological defects, and their flagella were immotile (Figures 2D–2F). In contrast, the heterozygous males were fertile, although their sperm flagella were often mildly swollen, bent irregularly at the annulus, or both. Females with heterozygous or *Hoatz*^*−/−*^ mutations were fertile (average litter sizes: 7.2 ± 2.8, n = 24, and 7.1 ± 1.9, n = 8 for heterozygous and *Hoatz*^*−/−*^ mice, respectively), although they exhibited higher mortality rates owing to obstructed labor compared with that of wild-type (0 of 22, 4 of 13, and 2 of 8 crossings for wild-type, heterozygous, and *Hoatz*^*−/−*^ females, respectively).

The deletion of *Hoatz* was not embryonic lethal, because *Hoatz*^*−/−*^ mice generated from crosses with the heterozygotes were born at the expected Mendelian frequency (Figure S2H). The *Hoatz*^*−/−*^ mice did not display laterality defects, polydactyly, polycystic kidney, or other notable abdominal organ abnormalities, indicating that the nodal and primary cilia were unaffected.

### Motile Cilia Are Variably Affected by the Hoatz^−/−^ Mutation

**Figure 3:**
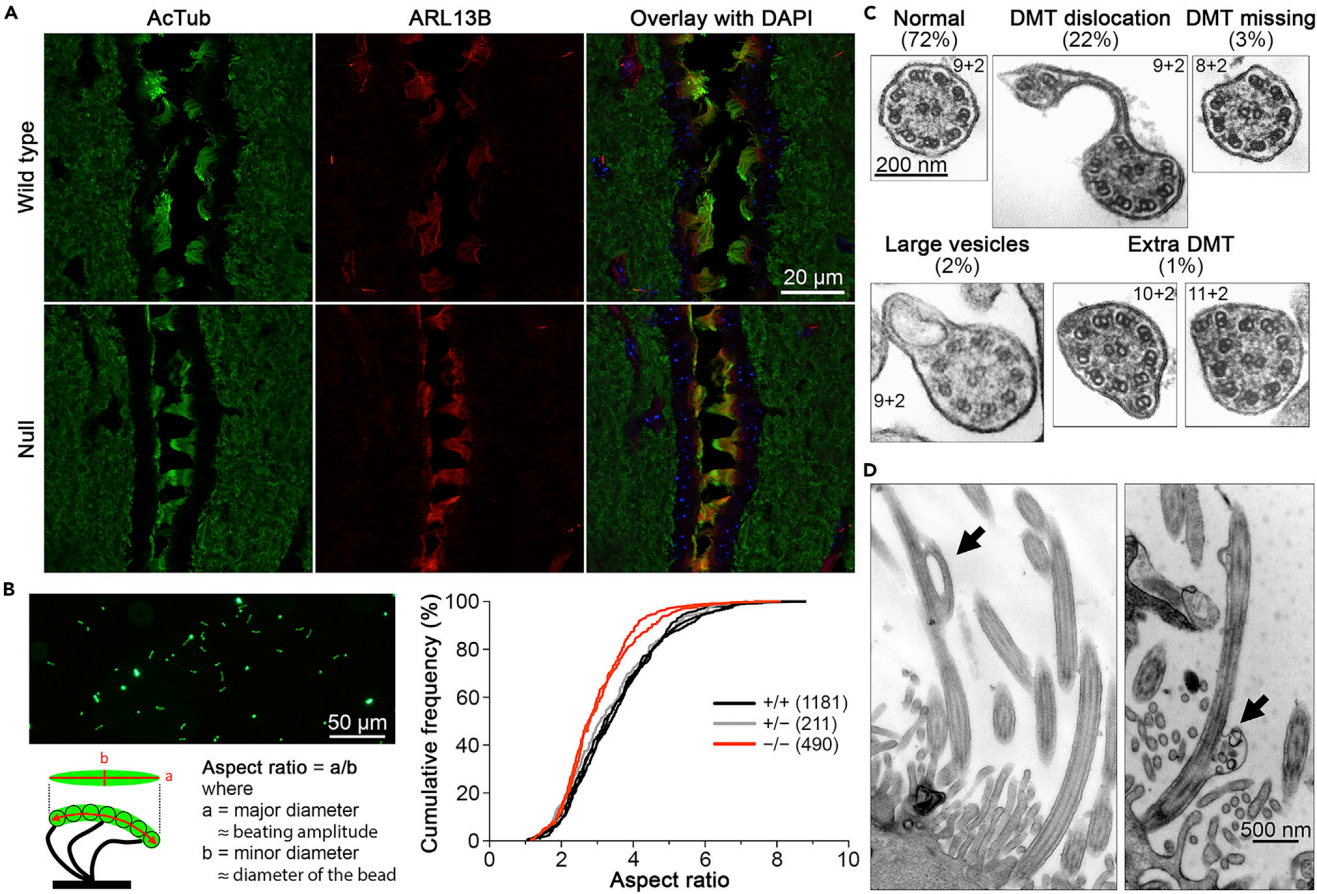
Motile Cilia Are Variably affected by the *Hoatz*^*−/−*^ Mutation (A) Confocal microscopy of the third ventricle of wild-type and *Hoatz*^*−/−*^ mice. These coronal brain sections were incubated with antibodies against acetylated α-tubulin (green) and ARL13B (red). The nuclei were detected using DAPI (blue). Note the collapsed and straight mutant cilia. Scale bar, 20 μm. (B) Measurement of the ciliary beating amplitude of primary cultured ependyma. Top left: representative image. The movement of motile cilia was visualized using fluorescent microbeads (exposure time, 50 ms). The major and minor axes of the best-fitting ellipse to the trace were calculated using ImageJ. Bottom left: The beating amplitude was estimated as the value of the aspect ratio (major diameter)/(minor diameter) assuming that the minor axis represents the diameter of the microbead. Right: Cumulative frequency plot of the aspect ratios of wild-type (n = 1,181), heterozygotes (n = 211), and null mutants (n = 490). p < 0.001, *Hoatz*^*−/−*^ versus wild-type. (C) Representative horizontal sections of ependymal cilia observed in *Hoatz*^*−/−*^ mice. The cilia (n = 216) were classified into five groups. DMT, doublet microtubules. (D) Representative longitudinal sections of the motile cilia of the ependyma of a *Hoatz*^*−/−*^ mutant mouse showing a dislocated axonemal microtubule or an abnormal vesicle-containing small particle (black arrows). See also Figure S3.

Immunohistochemical analysis of brain sections for the expression of acetylated α-tubulin (AcTub) and ADP-ribosylation factor-like 13B (ARL13B) detected some *Hoatz*^*−/−*^ ependyma with very short or collapsed cilia, suggesting a partial defect in the generation or maintenance of cilia (Figure 3A). Furthermore, the mutant cilia appeared erect, suggesting defects in motility. To identify the nature of the motility defect, we performed fluorescence imaging of ependyma on glass-bottom dishes. The beating amplitudes of motile cilia of *Hoatz*^*−/−*^ mutant cilia were smaller compared with those of wild-type and heterozygous mice (Figure 3B). Consistent with these observations, when the motility of ependymal cilia was investigated using high-speed video microscopy, the amplitude of ciliary beating (Video S1) and the beating frequency (Figure S1C) were lower compared with those of the wild-type. Stable expression of HOATZ-FLAG rescued these motility defects of the *Hoatz*^*−/−*^ ependyma.

Video S1. High-Speed Video Microscopy of Cultured Ependyma, Related to Figure 1 The images were recorded at 175 frames/s and viewed at 17.5 frames/s.

We next used transmission electron microscopy to observe horizontal sections of ependymal cilia of the brain ventricles. Although the majority (72%) had an apparently normal structure, defects in the arrangement of the axonemal microtubules were observed in the others (Figure 3C). The most frequent defect was outer doublet microtubule dislocations, in which one or two outer doublets were displaced from the correct position (22%). Other structural defects observed at lower frequencies were lack of one doublet microtubule (3%) and presence of extra doublet microtubules (1%). Some cilia exhibited an empty vesicle-like structure just beneath the ciliary membrane (2%); however, such vesicles were observed in the wild-type with similar frequencies and may therefore be unrelated to those of the *Hoatz*^*−/−*^ (Figure S3A). In all cases, the central pair appeared intact. In contrast to the abnormality in the axoneme, the structures of the basal bodies, as well as the ciliary transition zone, had no apparent defects (Figure S3B). When the longitudinal sections were investigated, we observed abnormal cilia with a partial dislocation of outer doublet microtubules, consistent with the above observation (Figure 3C), or a vesicle containing heterogeneous particles (Figure 3D). Together, these data demonstrate that the mutant ependymal cilia had structural defects in the outer doublet microtubules, suggesting that this may cause a reduced beating amplitude.

**Figure 4:**
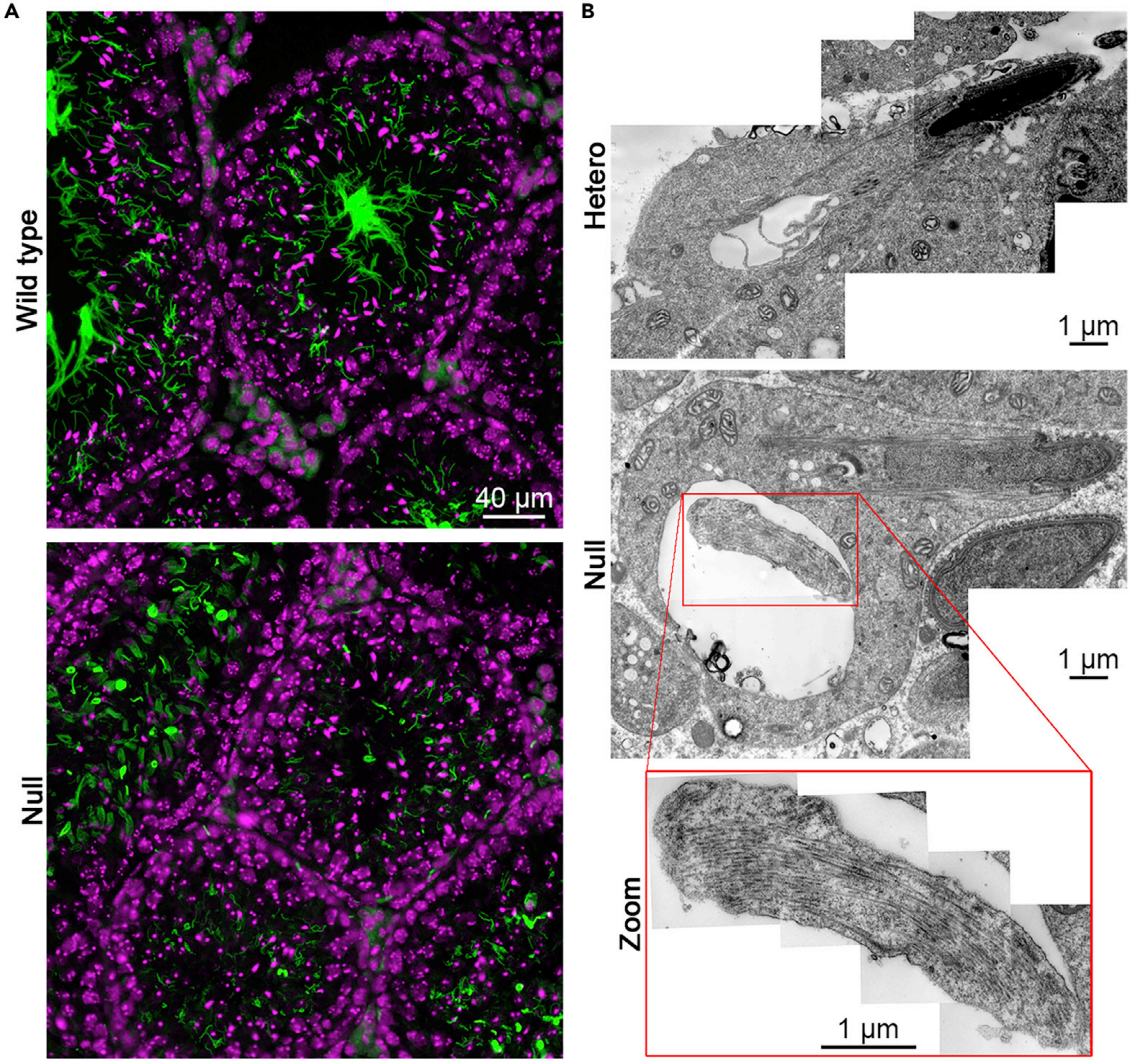
*Hoatz*^*−/−*^ Seminiferous Tubules Exhibit Structural Defects (A) Representative fluorescence microscopy images of the testes from wild-type and *Hoatz*^*−/−*^ mice. The sections were subjected to immunohistochemistry to detect acetylated α-tubulin (green). Nuclei were detected using DAPI (pseudocolored magenta). Scale bar, 40 μm. (B) Representative transmission electron microscopy (TEM) image tiling of spermatids undergoing cytodifferentiation in the asymptomatic heterozygous and *Hoatz*^*−/−*^ mutants. In the *Hoatz*^*−/−*^ mutant, a deformed flagellum contained an abnormal bundle of filaments (magnified). See also Figures S4 and S5.

We next conducted immunohistochemical analyses to identify structural defects of sperm flagella. Expression of AcTub by the *Hoatz*^*−/−*^ mutant testis showed the lack of fully developed flagella in the lumen of the seminiferous tubules (Figure 4A). We adopted a semiquantitative numerical scoring system recommended by the OECD guidelines for the histopathological evaluation of the male reproductive system (Creasy, 2008). We assigned grade 5 (severe), because the *Hoatz*^*−/−*^ testes lacked detectable luminal flagellar bundles in all seminiferous tubules (0 of 408 tubules, n = 4 sections), in contrast to wild-type (295 of 312 tubules, n = 2 sections). Although short flagella (approximately ≤20 μm) were present, the number and the fluorescence intensity were significantly less compared with those of wild-type. Instead, brush-like structures, presumed to be the manchette (Lehti and Sironen, 2016), as well as curled aggregates, exhibited strong staining.

Transmission electron microscopy of the seminiferous tubules demonstrated that the flagella of the *Hoatz*^*−/−*^ mutant spermatids undergoing cytodifferentiation often had abnormal fibrous materials with a diameter (approximately 24 nm) resembling that of singlet microtubules, instead of the well-organized axoneme (Figures 4B and S4). Accessory structures such as outer dense fibers, the fibrous sheath in the principal piece, and the spiral mitochondria in the midpiece were infrequently observed. Instead, many abnormal vesicles were observed in the cytoplasm. In early-phase spermatids with round nuclei, long developing flagella with an apparently intact axoneme without accessory structures were detected (data not shown), consistent with the immunohistochemical analysis that detected some short flagella. Together, these ultrastructural abnormalities indicate that the mutant spermatids elongated but did not maintain the axoneme, leading to severe destruction of the flagella.

The ultrastructures of motile cilia in the tracheal epithelia were investigated. Unlike those in ependyma or spermatids, the mutant axoneme was intact (Figure S5). Occasionally, blebs of the ciliary membrane were observed in distal sections.

### HOATZ Interacts with Enolase 4

**Figure 5:**
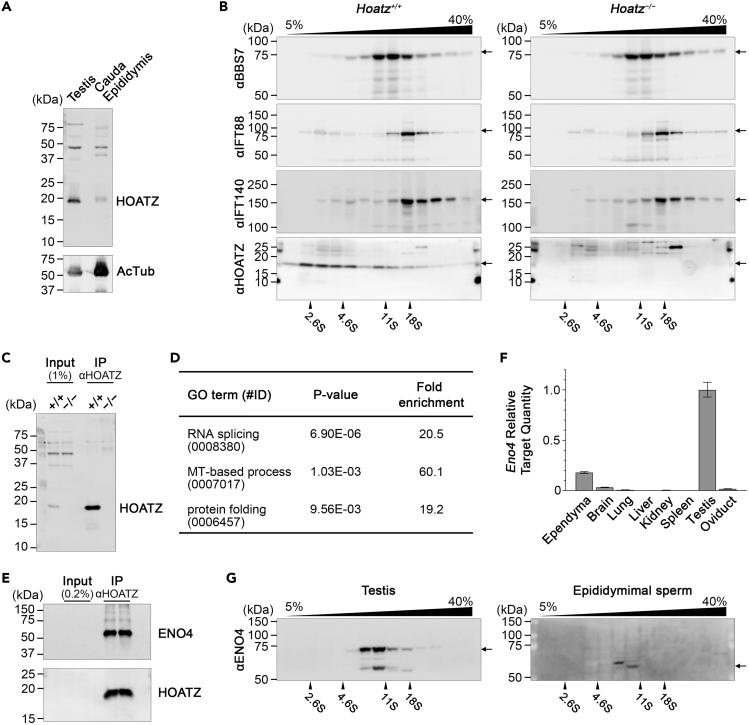
HOATZ Interacts with Enolase 4 (A) Comparison of endogenous HOATZ levels between testis and cauda epididymis containing mature sperm in wild-type mice. AcTub served as a marker of cilia and flagella. (B) Comparison of BBS7, IFT88, and IFT140 levels between wild-type and *Hoatz*^*−/−*^ mouse testes. Tissue homogenates were fractionated using sucrose density gradient centrifugation and concentrated using TCA precipitation. The levels of BBS7, IFT88, IFT140, and HOATZ were analyzed using western blotting with the respective antibodies. (C) Immunoprecipitation for shotgun proteomics. Testis homogenates from wild-type and *Hoatz*^*−/−*^ mice were incubated with a rabbit polyclonal anti-HOATZ IgG cross-linked to magnetic beads, and the eluates were subjected to LC-MS to detect potential HOATZ-binding proteins. HOATZ levels in the eluates were determined using western blotting. (D) Gene Ontology analysis of proteins eluted from of the anti-HOATZ immunoprecipitates. A dataset comprising 27 HOATZ-associated proteins was analyzed using the DAVID server (Huang et al., 2009). (E) Western blot analysis of ENO4 eluted from immunoprecipitates. (F) Real-time PCR analysis of *Eno4* mRNA levels using the comparative C_T_ method. The ΔΔC_T_ values were calculated using *B2m* as an endogenous reference and testis as a calibrator. The data are expressed as the mean, minimum, and maximum relative target quantities (n = 3). (G) Western blot analysis of ENO4 levels in the homogenates of testis and cauda epididymis containing mature sperm. See also Figure S6 and Data S1 and S2.

Having confirmed the abnormalities in the ependymal cilia and sperm flagella of the *Hoatz*^*−/−*^ mutant, the molecular function of HOATZ was investigated. To determine if HOATZ serves as a structural component of mature sperm flagella that confers structural integrity, we determined the levels of HOATZ and AcTub expressed by wild-type testes and cauda epididyma (Figure 5A). AcTub was detected at a high level in an epididymal lysate (Figure 5A), consistent with the massive amount of mature sperm stored in the duct (Wang, 2003). In contrast, HOATZ was predominantly expressed in the testis, but at significantly lower levels in the epididymis, consistent with those of intraflagellar transport (IFT) proteins (San Agustin et al., 2015). These data indicate that HOATZ is not a structural component of sperm flagella and may contribute to the formation of motile cilia and flagella.

To investigate the effects of the *Hoatz* knockout on the ciliogenesis machinery of the Bardet-Biedl syndrome complex (BBSome) and the IFT complex, testes from the wild-type and *Hoatz*^*−/−*^ mice were lysed, fractionated using sucrose density gradient centrifugation (Nachury et al., 2007), and subjected to western blot analyses to detect BBS7, IFT88, and IFT140. The levels and integrities of the BBSome, IFT-B, and IFT-A complexes were unaffected in the absence of HOATZ (Figure 5B). Furthermore, immunoprecipitation analysis of wild-type lysates failed to detect a direct interaction between HOATZ and BBS7 or IFT88 (data not shown).

In the testis homogenate, HOATZ (19 kDa) sedimented significantly faster through a sucrose gradient compared with chymotrypsinogen A (2.6S, 25 kDa) and was distributed over a broad range of fractions, including those containing apoferritin (17.6S, 490 kDa) (Figure 5B). In contrast, when a whole-cell lysate of 293T cells overexpressing recombinant HOATZ-FLAG was similarly analyzed, ectopically expressed HOATZ was enriched at the top of the gradient as expected according to its molecular mass, suggesting it existed as a free monomer (Figure S6A). These data suggest that HOATZ associated with tissue-specific proteins, likely those involved in motile ciliogenesis.

To identify the binding partners of HOATZ, testis lysates were immunoprecipitated with the anti-HOATZ antibody (Figure 5C). Liquid chromatography-mass spectrometry identified 253 and 260 proteins in the wild-type and the *Hoatz*^*−/−*^ mutant samples, respectively (Data S1). We selected for further study those that were specifically or more frequently (>3 fold-higher peptide-to-spectrum matches) detected in the wild-type sample. In the resulting new dataset containing 27 proteins, HOATZ (C11ORF88 homolog) was ranked as the second most frequently detected protein (Data S2). We detected the tubulins TUBB5, TUBB3, and TUBA3A; heat shock proteins HSPA8, HSPA1L, and HSPA4L; and deleted in lung and esophageal cancer protein 1 (DLEC1, also known as CFAP81) as candidate HOATZ-binding proteins. We also detected RNA-binding proteins such as apoptotic chromatin condensation inducer 1 (ACIN1), RNA binding protein with serine rich domain 1 (RNPS1), DEAH-box helicase 15 (DHX15), small nuclear ribonucleoprotein U4/U6.U5 subunit 27 (SNRNP27), and arginine and serine rich coiled-coil 1 (RSRC1) whose tissue expression patterns and functions do not correlate with motile cilia and flagella. Gene Ontology analysis of these proteins suggested that HOATZ is involved in the biological processes of RNA splicing, microtubule-based process, and protein folding (Figure 5D). This dataset included metabolic enzymes involved in the pay-off phase of glycolysis, enolase 4 (ENO4) and glyceraldehyde-3-phosphate dehydrogenase (GAPDH) (Data S2). To assess an interaction between HOATZ and ENO4, testis lysates were immunoprecipitated using the anti-HOATZ antibody as described above, and the eluates were subjected to western blot analysis. As we expected, ENO4 was enriched in the eluate along with HOATZ (Figure 5E).

We further studied ENO4, because male *Eno4* KO mice are infertile because of a severe defect of spermatogenesis, similar to that of the *Hoatz*^*−/−*^ phenotype described above (Nakamura et al., 2013). Real-time PCR analysis confirmed that *Eno4* mRNA was expressed at high levels in testis, to some extent in the ependyma (Figure 5F), but at low levels in lung and oviduct. Western blot analysis of wild-type tests homogenates fractionated using sucrose density gradient centrifugation demonstrated that ENO4 was enriched in fractions of approximately 10S, overlapping only partially with HOATZ (Figure 5G). The difference in the distributions of ENO4 and HOATZ in the sucrose density gradient suggested that these proteins may not exclusively associate. Furthermore, western blotting detected a major band at approximately 70 kDa and a minor band at approximately 60 kDa. In contrast, the 70-kDa band was not detected in wild-type cauda epididyma, although the 60 kDa was (Figure 5G). We therefore speculated that ENO4 may undergo processing before its transport to mature flagella.

### HOATZ Mediates the Processing of ENO4

**Figure 6:**
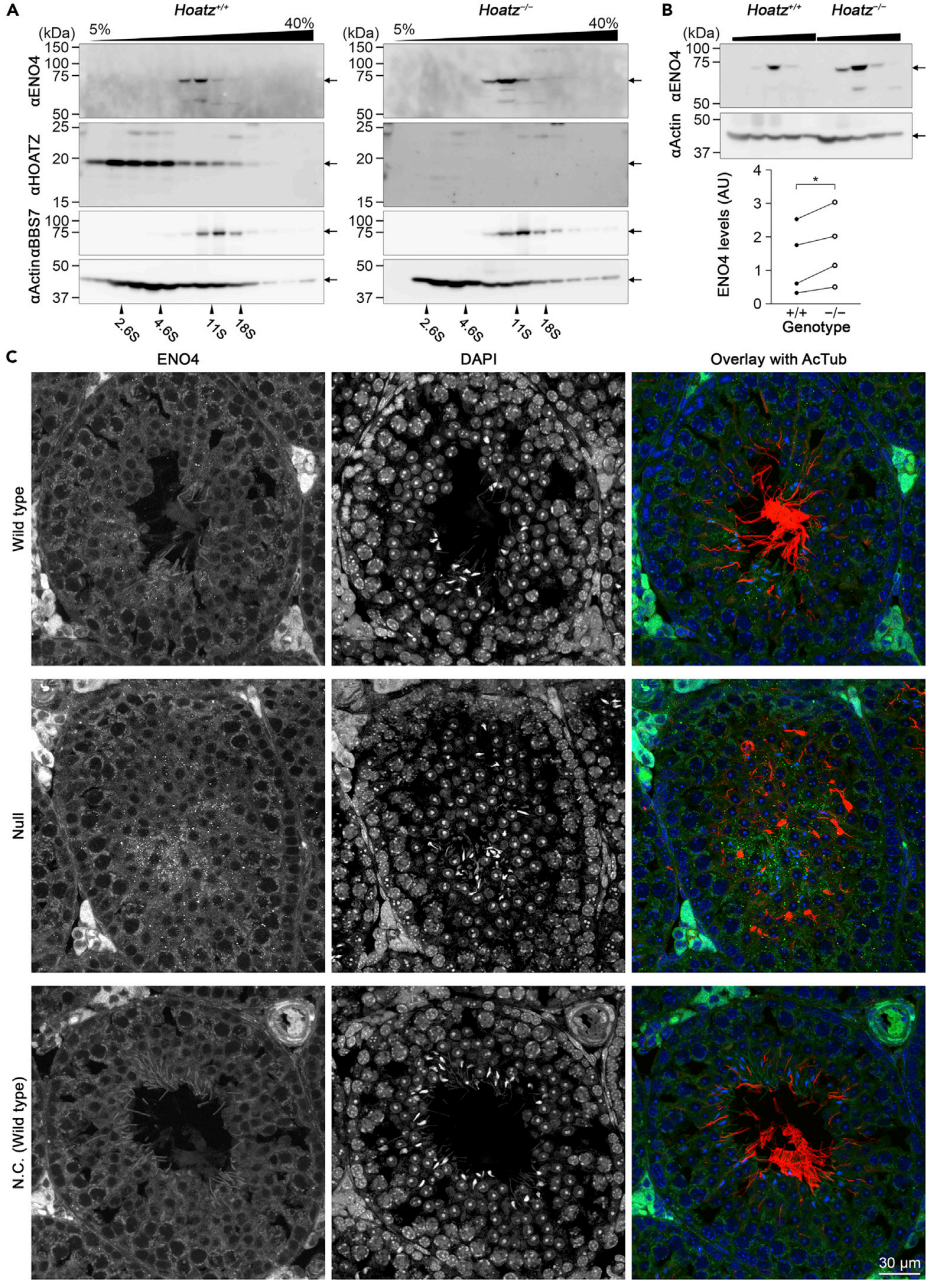
Abnormal Accumulation of ENO4 in the Testes of *Hoatz*^*−/−*^ Mice (A) Western blot analysis of ENO4 levels in wild-type and the *Hoatz*^*−/−*^ mouse testes. The tissue homogenates were fractionated using sucrose density gradient centrifugation, and the levels of ENO4, HOATZ, BBS7, and actin isoforms (loading control) were determined. (B) Top: Fractions containing ENO4 from wild-type and *Hoatz*^*−/−*^ mice were resolved on the same gel. Bottom: Quantification of the immunoreactive bands. The data are expressed as relative values (arbitrary unit) to actin (paired t test, ∗p = 0.02, n = 4). (C) Representative confocal images of stages V–VII seminiferous tubules, ENO4 (green), AcTub (red), and DAPI (blue). As a negative control (N.C.), a wild-type section was incubated with normal rabbit IgG and mouse anti-AcTub. Note the accumulation of ENO4-positive puncta at the luminal side of the *Hoatz*^*−/−*^ seminiferous tubule. Scale bar, 30 μm. See also Figure S6.

Western blot analysis of sucrose density gradients showed an increase of ENO4, particularly that of its 70-kDa form, in the *Hoatz*^*−/−*^ mutant (Figure 6A). To directly compare ENO4 levels between the *Hoatz*^*−/−*^ and wild-type mice, the ENO4-positive fractions from both genotypes were loaded on the same gel and blotted on the same membrane to compare their signal intensities (arbitrary units) relative to that of an internal standard (actin) (Figure 6B). The differences were statistically significant (p = 0.02, n = 4). When the testes sections were subjected to immunohistochemical analysis, ENO4 was detected in small puncta in the cytoplasm of haploid spermatids of the wild-type and mutant. Furthermore, the tubules of the *Hoatz*^*−/−*^ mutant exhibited many puncta compared with those of the wild-type during seminiferous stages V–VII (Figure 6C). Similarly, when primary cultured ependyma were homogenized and fractionated on sucrose density gradients, western blot analysis detected an increase in ENO4 levels in the *Hoatz*^*−/−*^ mutant (Figure S6B), strongly suggesting that HOATZ mediated the maturation of ENO4.

## Discussion

**Figure 7:**
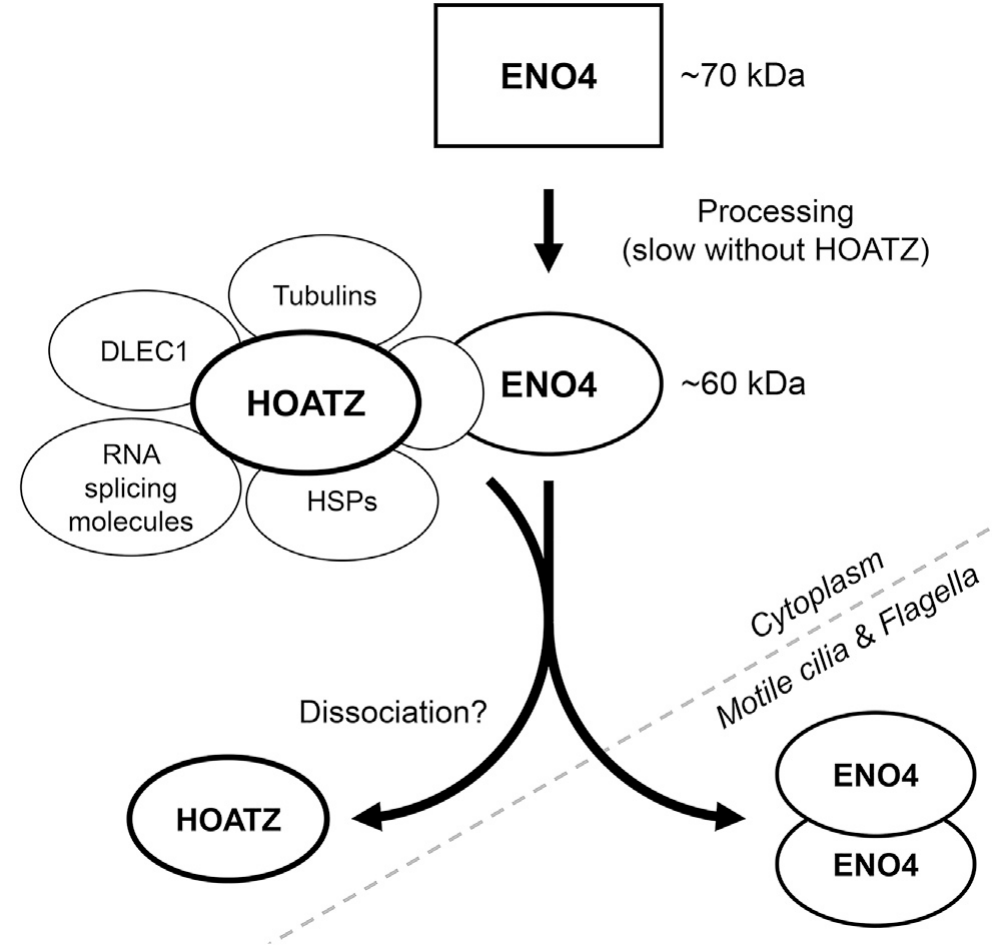
Diagram Showing the Proposed Function of HOATZ ENO4 is synthesized as an approximately 70-kDa precursor, which then undergoes proteolytic cleavage to an approximately 60-kDa enzyme. HOATZ associates directly or indirectly with ENO4 to mediate this process.

Here our characterization of the cilia-related gene *Hoatz* (*4833427G06Rik*) sheds light on the molecular mechanism of motile ciliogenesis that is particularly important in spermatids and the ependyma. *Hoatz* was first identified as one of 99 mouse cilia-related genes, according to its tissue expression pattern (McClintock et al., 2007). Microarray analysis shows that in zebrafish, *hoatz* (*C11orf88* homolog) is among the top 15 upregulated genes in testis (Small et al., 2009). Our RT-PCR data (Figure 1) are consistent with these reports. *Hoatz* is a candidate *Foxj1*–dependent factor in the lung, but not for *Noto* effectors in the embryonic node (Stauber et al., 2017), which agrees with our observation that *Hoatz*^*−/−*^ mice had no detectable L-R defect. However, *Hoatz*^*−/−*^ mice exhibited severe defects in spermatogenesis, leading to infertility and varying degrees of hydrocephalus (Figures 2, 3, and 4). The results of our present biochemical analyses (Figures 5 and 6) lead us to propose that HOATZ mediates the maturation of the glycolytic enzyme ENO4, thus contributing to the translocation of the latter during motile ciliogenesis and flagellar genesis (Figure 7).

Enolase catalyzes the conversion of 2-phosphoglycerate to phosphoenolpyruvate in the pay-off phase of glycolysis. Vertebrate genomes encode *Eno1*–*4*. *Eno1* is expressed ubiquitously, and *Eno2* and *Eno3* are specifically expressed in neurons and muscles (skeletal and heart), respectively (Isgrò et al., 2015, Merkulova et al., 1997), suggesting the importance of the latter two isoforms in ATP production in highly energy-demanding cell types. Similarly, *Eno4* is specifically expressed by spermatogenic cells and has been associated with a previously characterized sperm-specific enolase activity of ENO-S (Edwards and Grootegoed, 1983, Nakamura et al., 2013). Here, the expression of *Eno4* mRNA and protein was detected in spermatids and ependyma (Figures 5, 6, S6B). Thus, these cells may serve as a third energy-demanding cell type that requires specific enolases. Although previous proteomic analyses of mature human sperm detected ENO1 (Martínez-Heredia et al., 2006) and ENO4 (Vandenbrouck et al., 2016), which may indicate functional redundancy and compensation, the KO phenotype of *Eno4* mice demonstrates the requirement of *Eno4* in the formation of sperm flagella formation (Nakamura et al., 2013).

*Eno4* and *Hoatz* are conserved only in vertebrates, indicating that they may have been acquired to accomplish motile ciliogenesis specific to vertebrates. Interestingly, spermatids and ependyma reside in anatomical regions protected by the blood-testis and blood-brain barriers, respectively, indicating their requirement in tissues in which the nutrient supply from circulating blood is strictly regulated. However, although ENO1–3 share the same catalytic site structure, which is evolutionarily conserved (López-López et al., 2018), ENO4 has several substitutions in those critical residues, raising concerns about the enolase activity (K. Narita, Unpublished Data). As enolases form homo- and heterodimers (Ueta et al., 2004), ENO4 may heterodimerize with ENO1 to act together with HOATZ as its transporter into motile cilia and flagella. Furthermore, ENO4 may bind 2-phosphoglycerate to protect this high-energy substrate from degradation. In addition, functions other than glycolysis are associated with ENO1 protein and its fragment (Ji et al., 2016, Lung et al., 2010). Similarly, ENO4 may mediate multiple functions to participate in motile ciliogenesis independent of glycolytic activity.

Interestingly, although *Eno4* KO mice exhibited severe malformations of the spermatids of the cauda epididymis, only minor abnormalities were apparent in the histology and ultrastructure of the testis, with no indication of hydrocephalus (Nakamura et al., 2013). Although the highly analogous morphological defects of the *Hoatz*- and *Eno4*-null spermatids support the conclusion that they function in the same biological process, the KO phenotype of the former was more severe, suggesting that the function of HOATZ is not limited to processing ENO4. The potential HOATZ-interacting proteins identified here (Data S2) suggest the involvement of HOATZ in RNA splicing, microtubule-based processes, and protein folding. These possibilities will be addressed in the future.

In *Chlamydomonas* flagella, axonemal enolase forms the central pair complex CPC1, a component of the C1b projection, together with HSP70A and other proteins (Mitchell, 2005). Furthermore, recent proteomic analysis of the central pair of *Chlamydomonas* flagella identified DLEC1-homolog FAP81 as a component of the C1a projection (Zhao et al., 2019). The amino acid sequences of *Chlamydomonas* enolase are 67% and 26% identical to those of mouse ENO1 and ENO4, respectively, and our present shotgun proteomic analysis identified enolase, heat shock proteins, tubulins, and DLEC1 as potential HOATZ-interacting proteins (Data S2). Although our ultrastructural analysis of the *Hoatz*^*−/−*^ mutant ependymal cilia demonstrates instabilities of the outer doublets rather than the central pair (Figure 3), HOATZ may contribute to the formation of the central apparatus in vertebrates. Clearly, further investigation is required to identify the functional relationships among ENO1, ENO4, and HOATZ that mediate vertebrate motile ciliogenesis.

Our present data demonstrate that *Hoatz* is a ciliopathy gene of mice. On the other hand, there are no reports of mutations in *HOATZ* that are associated with human diseases. This may be explained by the small size of the exons, which decreases the probability of random mutagenesis and its autosomal recessive mode of inheritance. Similar to *HOATZ*, other uncharacterized small genes may have significant biological functions that are not associated with human pathology (Pauli et al., 2014). Moreover, small genes encoding proteins comprising fewer than 100 amino acid residues are difficult to annotate (Pauli et al., 2015). Recent advances in the genome editing technology promise to enhance our understanding of small, nonannotated genes.

### Limitations of the Study

We are checking a presumed null allele with a novel antibody and consider this as a limitation of the study. The specificity of the antibody has been confirmed using the strain #3. The strains #1 and #2 had been cryopreserved using heterozygous sperm because of the cost required to maintain the mice.

## Methods

All methods can be found in the accompanying Transparent Methods supplemental file.

## Data and Code Availability

The datasets supporting the current study have not been deposited in a public repository because of large file size but are available from the corresponding author on request.

## Supplemental Information

Document S1. Transparent Methods and Figures S1–S6

Data S1. List of Proteins Identified by LC-MS Analysis, Related to Figure 5 This Excel file contains two datasheets showing the results obtained from the wild-type and *Hoatz*^*−/−*^ samples.

Data S2. Candidate HOATZ-Interacting Proteins, Related to Figure 5 The proteins detected in the wild-type and *Hoatz*^*−/−*^ samples (shown in Data S1) were compared to identify those specifically detected or more frequently (>3-fold higher peptide-to-spectrum matches) in the former. HOATZ is indicated in red. The proteins highlighted in blue, green, and orange are associated with enriched GO terms of RNA splicing, microtubule-based process, and protein folding, respectively. The glycolytic enzymes are highlighted in pink.
