# Supplementary material for: Discovery of a Vertebrate-Specific Factor that Processes Flagellar Glycolytic Enolase during Motile Ciliogenesis
Source: iScience. 2020 Mar 19;23(4):100992. doi: 10.1016/j.isci.2020.100992 (PMC7132099; doi:10.1016/j.isci.2020.100992)
Supplement: Document S1. Transparent Methods and Figures S1–S6 [file mmc1.pdf]

iScience, Volume *23*

Supplemental Information

Discovery of a Vertebrate-Specific Factor that Processes Flagellar Glycolytic Enolase during Motile Ciliogenesis

Keishi Narita, Hiroaki Nagatomo, Hiroko Kozuka-Hata, Masaaki Oyama, and Sen Takeda

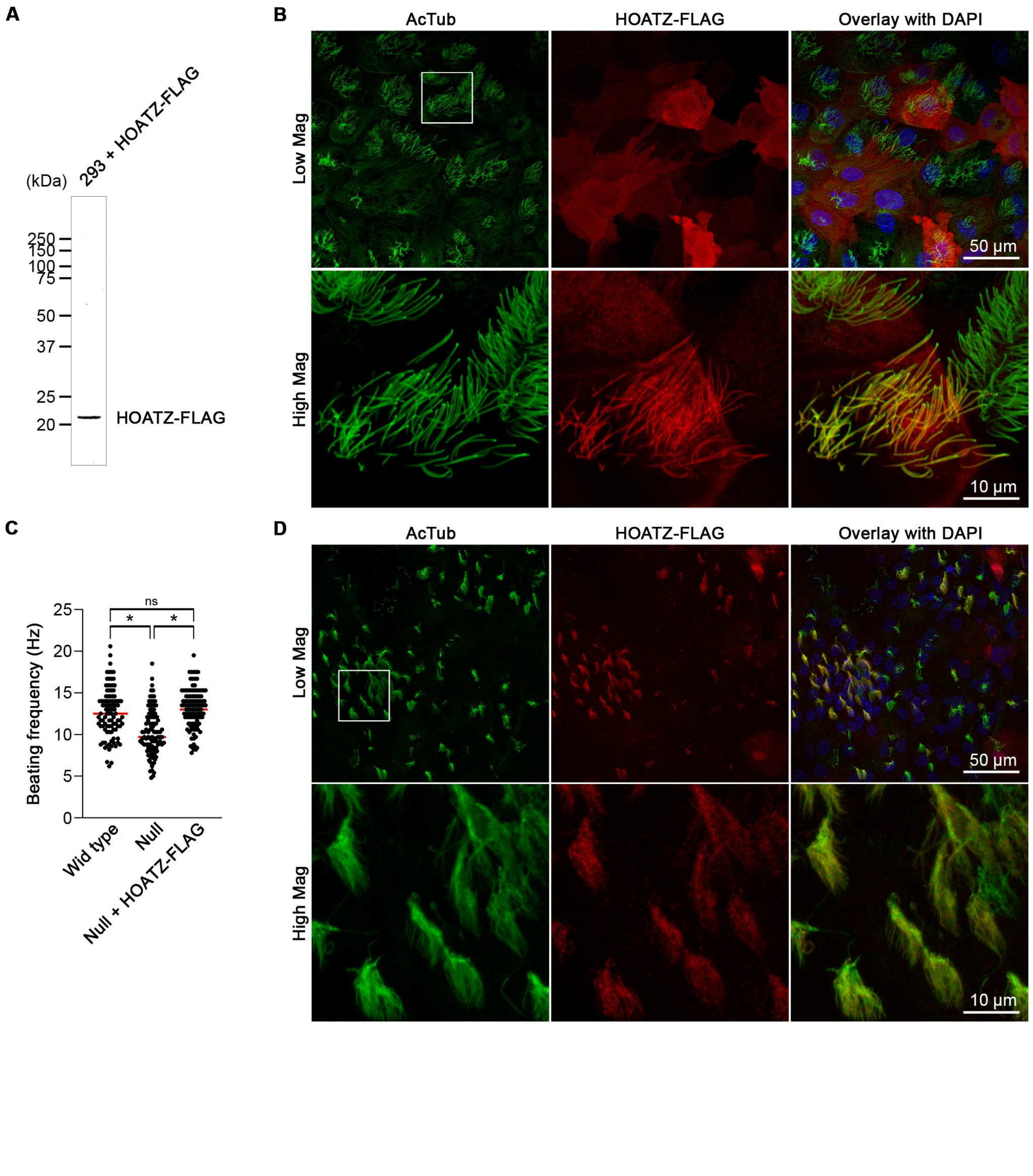
A
293 + HOATZ-FLAG
(kDa)
250
150
100
75
50
37
25
20
HOATZ-FLAG
B
AcTub
HOATZ-FLAG
Overlay with DAPI
Low Mag
High Mag
50 μm
10 μm
C
Beating frequency (Hz)
25
20
15
10
5
0
ns
*
*
Wid type
Null
Null + HOATZ-FLAG
D
AcTub
HOATZ-FLAG
Overlay with DAPI
Low Mag
High Mag
50 μm
10 μm

Supplemental Figure S1. Analysis of the subcellular localization of HOATZ-FLAG in cultured brain ependyma, Related to Figure 1. (**A**) Western blot analysis of HOATZ-FLAG expression. The proteins in a whole cell lysate of 293FT cells overexpressing HOATZ-FLAG were separated using a 5%–20% polyacrylamide gradient gel. The anti-FLAG antibody reacted with a protein that migrated at a position corresponding to 22 kDa (arrow). The positions and molecular masses (kDa) of the standards are indicated on the left. (**B**) Confocal fluorescence microscopy of cultured ependyma transduced with a lentiviral vector expressing HOATZ-FLAG under the control of the CMV promoter. The cells were analyzed for the expression of acetylated α-tubulin (green) and the FLAG epitope tag (red). Nuclei were detected using DAPI (blue). In the lower-magnification images, confocal sections were acquired from the base to the ciliary tip of the cells. In the higher-magnification images corresponding to the boxed area in the low magnification images, sections were limited to the apical space, including cilia. (**C**) Scatter plots with median lines showing the cilia beating frequencies of indicated cultured ependyma. High-speed video microscopy data were acquired in the same way as described for sperm flagellar beat frequency analysis. *P < 0.0001 between indicated groups (n = 100 cells for wild type, 103 for *Hoatz*$^{-/-}$, and 111 for *Hoatz*$^{-/-}$ + HOATZ-FLAG). (**D**) Confocal fluorescence microscopy of cultured *Hoatz*$^{-/-}$ ependyma transduced with a lentiviral vector expressing HOATZ-FLAG. The cells were analyzed for the expression of acetylated α-tubulin (green) and the FLAG epitope tag (red). Nuclei were detected using DAPI (blue).

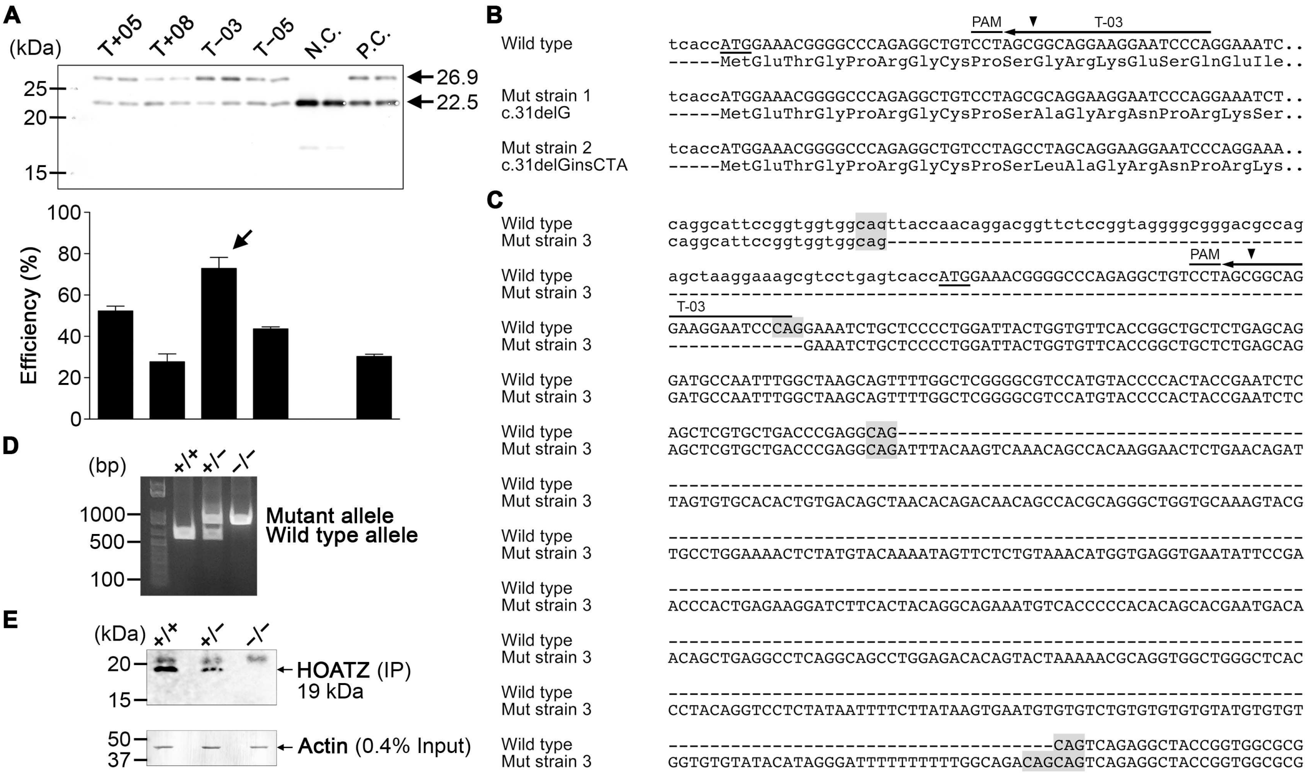

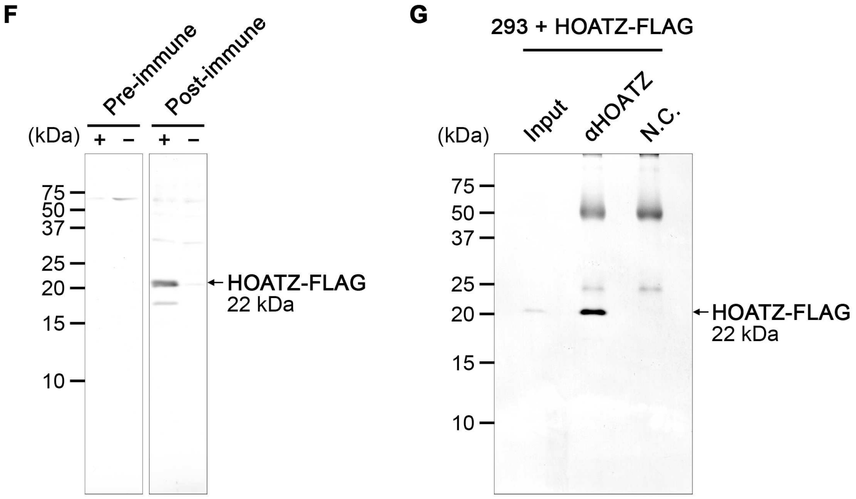

H

Genotype	Count	%
+/+	18	20
+/−	44	50
−/−	26	30

Supplemental Figure S2. Generation of *HOATZ*$^{-/-}$ mice using the CRISPR/Cas9 system, Related to Figure 2. (**A**) Quantitative analysis of the double-strand break efficiencies of pX330 clones targeting *Hoatz*. Top: Western blot analysis of 293T cells coexpressing one of the indicated pX330 clones (T+05, T+08, T−03, and T−05) and the pCAG-EGxxFP reporter construct containing the target region. The empty pX330 vector and the reporter construct served as negative controls, and the pX330 and pCAG-EGxxFP constructs for *Cetn1* (Mashiko et al., 2013) served as positive controls. The lower band (22.5 kDa) corresponds to the nonfluorescent fragment of EGFP encoded by the full-length pCAG-EGxxFP plasmid, whereas the 26.9-kDa band corresponds to full-length EGFP regenerated after the targeted double-strand break and subsequent repair processes. Bottom: The efficiencies of the pX330 clones were calculated according to the ratio of the intensities of each band, and values are expressed as the mean ± SEM (n = 2). (**B**) Nucleotide sequence analysis of *Hoatz* mutant strains #1 and #2. The position and direction of the T−03 guide RNA recognition sequence, the adjacent protospacer-adjacent motif (PAM), and the site of the targeted double-strand break are indicated on the wild-type reference sequence. (**C**) Nucleotide sequence analysis of *Hoatz* mutant strain #3. A relatively large deletion and insertion flanked by the CAG motif were introduced around the target site. (**D**) Genomic PCR analysis of strain #3. (**E**) Analysis of HOATZ expression in wild type, heterogeneous, and *Hoatz*$^{-/-}$ mouse testes at 10 weeks of age (strain #3). Whole testis extracts were immunoprecipitated using a rabbit anti-HOATZ polyclonal IgG, and the immunoprecipitates were analyzed using western blotting with the same anti-rabbit IgG. Rabbit TrueBlot (Rockland) was used as the secondary antibody to minimize the signal generated by IgG heavy and light chains. HOATZ (19 kDa) was undetectable in the *Hoatz*$^{-/-}$ mutant. A nonspecific band >20 kDa was present in all samples, which was a protein present in the testicular homogenate that bound to Protein G-Sepharose beads but was not detected when magnetic beads were used for immunoprecipitation. Pan-actin served as the loading control. (**F**) Specificity of the rabbit polyclonal anti-HOATZ antibody. Pre- and postimmune rabbit sera were used to probe a PVDF membrane containing proteins present in a whole cell lysate of 293T cells with (+) or without (−) expression of HOATZ-FLAG. The minor band (approximately 17 kDa) detected by the immune sera was a degradation product of recombinant HOATZ-FLAG. (**G**) Control immunoprecipitation experiment. HOATZ-FLAG overexpressed in 293-FT cells was immunoprecipitated using the rabbit anti-HOATZ antibody, resolved using a 15% polyacrylamide gel, and detected with the anti-FLAG antibody. Normal rabbit IgG served as a negative control. The faint bands present in the immunoprecipitates were the heavy and light chains of rabbit IgG. (**H**) Genotype distribution of offspring from heterozygous matings.

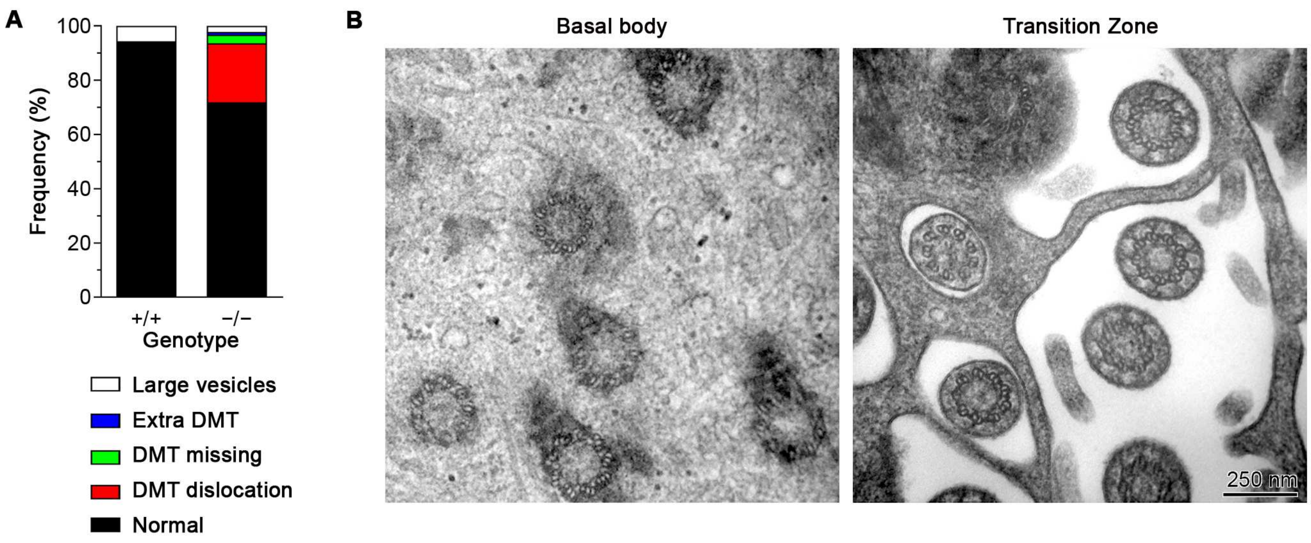
A
100
80
60
40
20
0
Frequency (%)
+/+
−/−
Genotype
Large vesicles
Extra DMT
DMT missing
DMT dislocation
Normal
B
Basal body
Transition Zone
250 nm

Supplemental Figure S3. Ultrastructural analysis of motile cilia in ependyma, Related to Figure 3. (**A**) Quantitative analysis of ciliary axoneme abnormalities. Horizontal sections of cilia of wild type (n = 387) and the *Hoatz*$^{-/-}$ mutant (n = 216) were inspected and classified into the indicated groups. (**B**) Representative TEM images of *Hoatz*$^{-/-}$ ependyma in the brain ventricle, showing apparently intact basal bodies and ciliary transition zones. Bar = 250 nm.

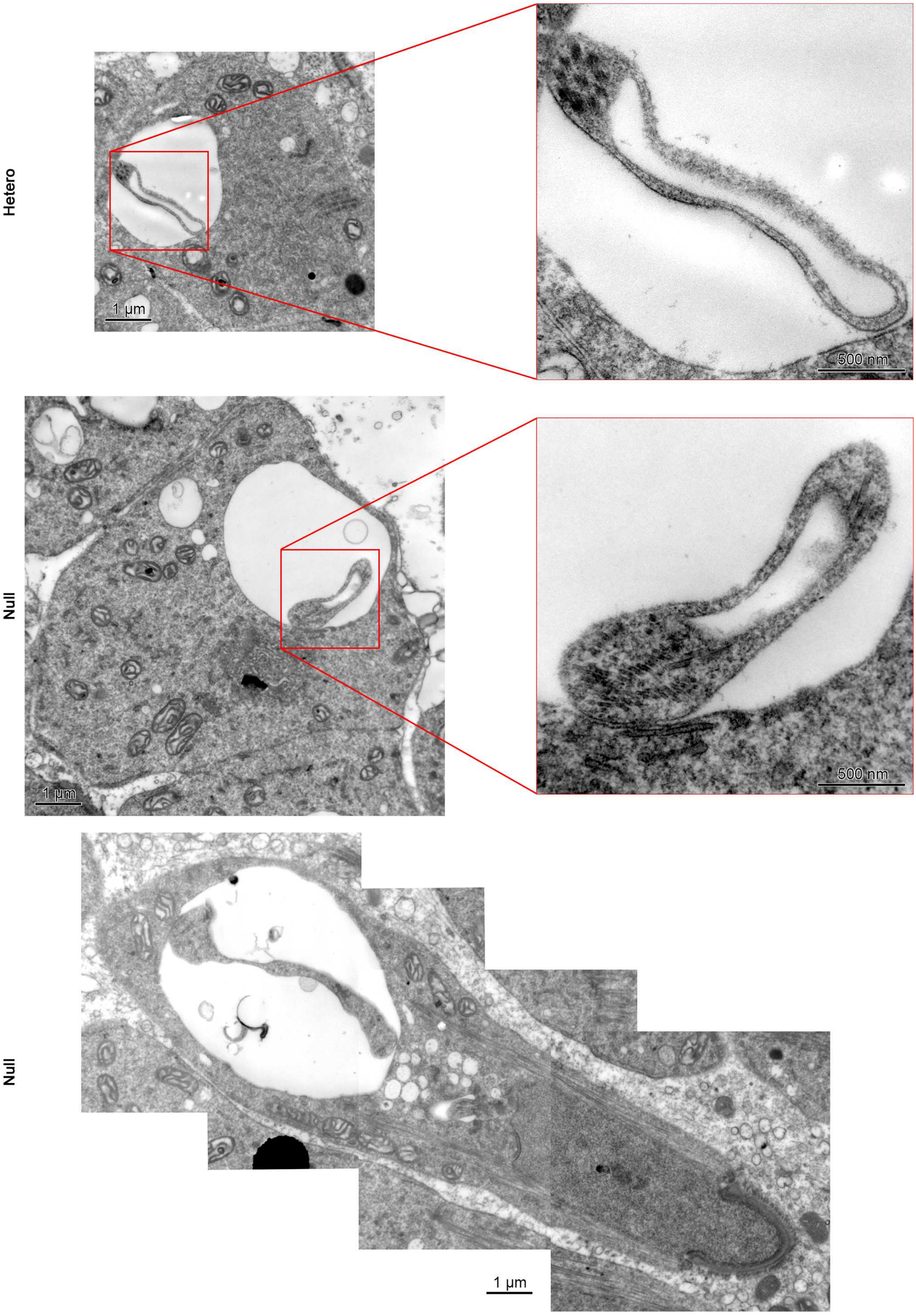

Hetero
1 µm
500 nm
Null
1 µm
500 nm
Null
1 µm

Supplemental Figure S4. Ultrastructural analysis of spermatids undergoing cytodifferentiation, Related to Figure 4. Representative TEM images of spermatids undergoing cytodifferentiation in the asymptomatic heterozygous and the *Hoatz*$^{-/-}$ mutants. In the *Hoatz*$^{-/-}$ mutant, a deformed flagellum contained a bundle of singlet microtubules (zoom). Abnormal intracellular vesicles were present in the cytoplasm.

A

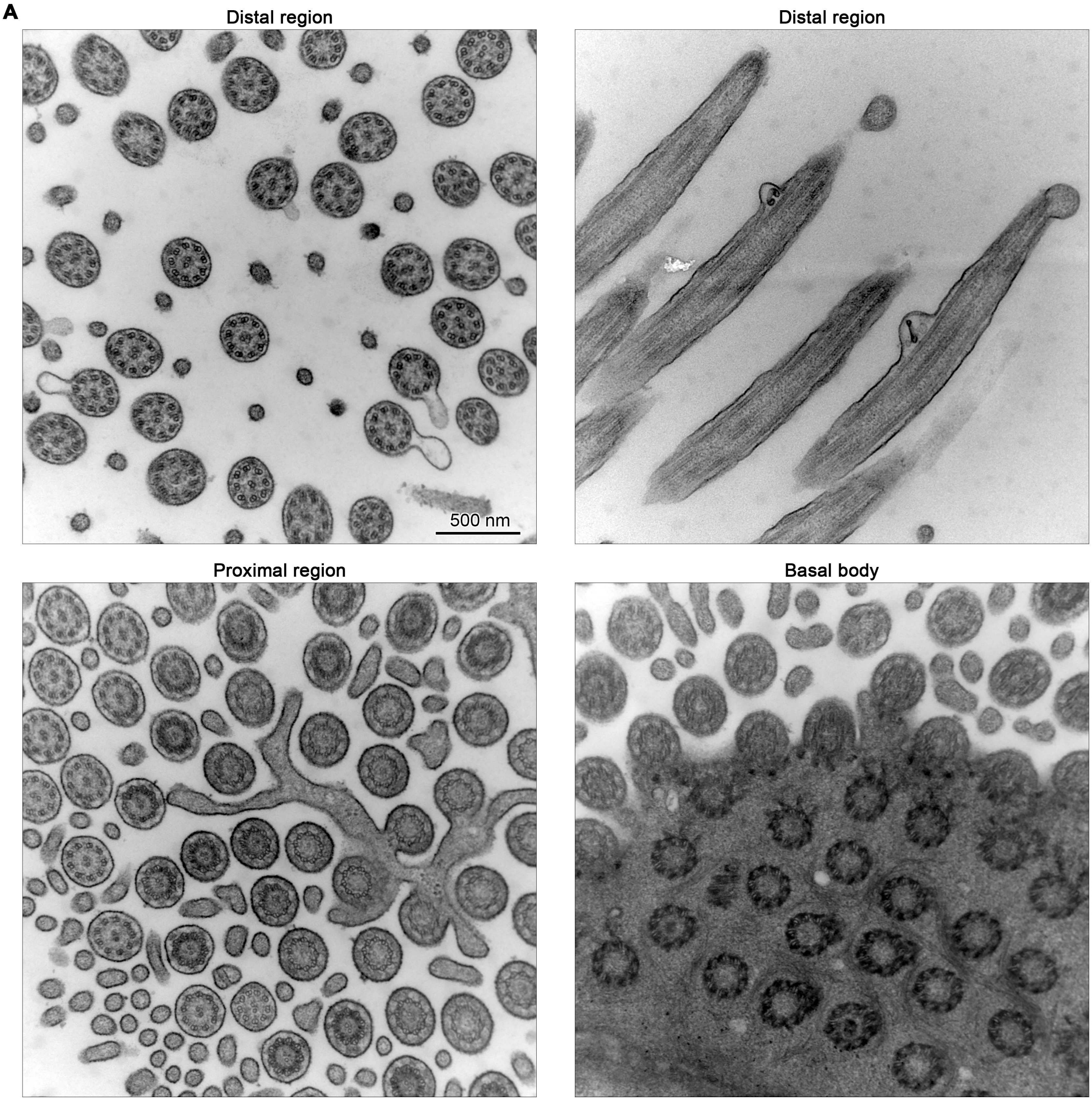

B

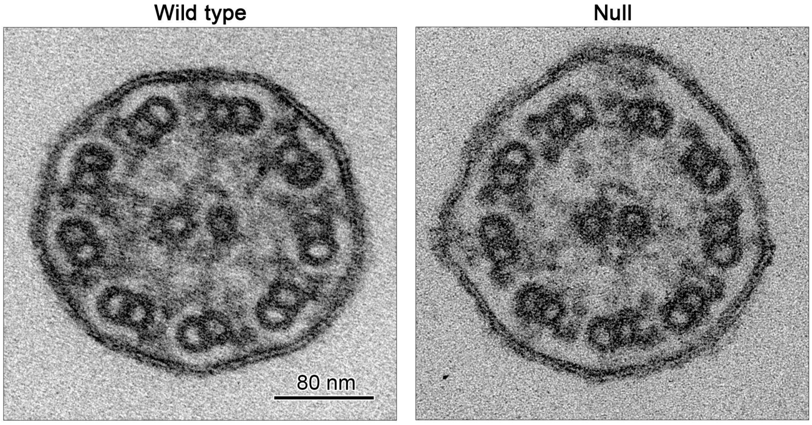

Microtubules	% (Null/WT)
Normal	95/95
Outer doublet defect	4/4
Central pair defect	0/0
Other defects	0/1

Dynein arms	% (Null/WT)
Normal	79/82
Outer arm defect	1/4
Inner arm defect	15/11
No arms	5/3

Supplemental Figure S5. Ultrastructural analysis of motile cilia in *Hoatz*$^{-/-}$ tracheal epithelia, Related to Figure 4. (**A**) Representative TEM images showing apparently intact structures. Bar = 500 nm. In the distal region of the cilia, there were several vesicle-containing swellings of axolemma, which represent ependymal cilia (Fig. 3C). (**B**) Representative higher magnification images of individual cross sections of cilia from wild type and *Hoatz*$^{-/-}$ trachea. The table summarizes the results of the quantitative analysis. For wild type control, 328 and 138 cilia were analyzed to score the structural integrity of 9+2 microtubules and the dynein arms, respectively. For *Hoatz*$^{-/-}$ mutant, 322 and 162 cilia were analyzed likewise. A detailed description of the analysis is provided in the Method section (Shoemark et al., 2012, 2020).

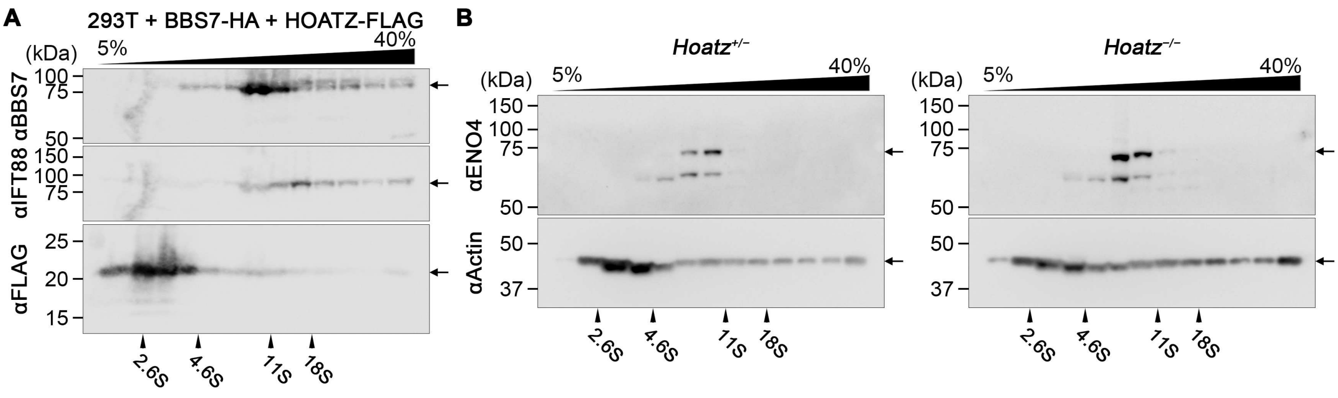

A
293T + BBS7-HA + HOATZ-FLAG
5%
40%
(kDa)
αBBS7
αIFT88
αFLAG
2.6S
4.6S
11S
18S
B
Hoatz+/−
Hoatz−/−
αENO4
αActin

Supplemental Figure S6. Abnormal accumulation of ENO4 in *Hoatz*$^{-/-}$ ependyma, Related to Figures 5 and 6. (**A**) Sucrose density gradient analysis of 293T cells overexpressing BBS7-HA and HOATZ-FLAG. Western blot analysis of the distributions of BBS7, IFT88 and recombinant HOATZ. (**B**) Western blot analysis of ENO4 levels of the asymptomatic heterozygous and *Hoatz*$^{-/-}$ mouse ependyma.

Transparent Methods

Mice and cell culture

The Institutional Animal Care and Use Committee at the University of Yamanashi approved the experiments using mice (Approval number: 29-43). C57BL/6J mice were from Charles River Laboratories Japan (Yokohama, Japan). Primary cultures of mouse ependyma were prepared from mechanically dissociated neonatal mouse cerebral hemispheres seeded on Matrigel-coated culture dishes (Narita et al., 2012). The cells were grown in Neurobasal-A medium containing B27 supplement (Thermo Fisher Scientific), while contaminating neurons were washed out by applying a stream of culture medium before changing the medium. Lenti-X 293T cells (Takara Bio, Shiga, Japan) and the 293FT cell line (Thermo Fisher Scientific, Waltham, MA) were cultured following the sources' instructions.

Molecular cloning, mammalian gene expression, and RNA in situ hybridization

The *Hoatz* cDNA was amplified from a cDNA library of cultured mouse ependyma using KOD Plus Neo DNA polymerase (Toyobo, Osaka, Japan) and the custom oligonucleotide primers (Thermo Fisher Scientific) with restriction sites indicated as follows: Hoatz BamHI Fwd (5′-ggatccATGGAAACGGGGCCCAGAGG-3′); Hoatz XhoI Rev (5′-ctcgagTTCCAAGGCTTTGACTTCTTCTTGGTCC-3′). The annealing temperature was 65 °C. The PCR product was cloned into the pGEM-T Easy vector (Promega, Madison, WI), and the sequence was confirmed. To express HOATZ-FLAG in mammalian cells, the *Hoatz* cDNA was cleaved from the cloning vector and ligated to the BamHI-XhoI sites of pCMV-Tag 4A (Agilent Technologies, Santa Clara, CA). For lentiviral expression of HOATZ-FLAG, the coding region of the pCMV-Tag 4A clone was amplified with KOD Plus Neo using the above BamHI Fwd and universal T7 promoter primers and then ligated to the Eco47III site of CSII-CMV-MCS-IRES2-Bsd (provided by Dr. Hiroyuki Miyoshi [RIKEN BioResource Center] along with the packaging plasmids pCAG-HIVgp and pCMV-VSV-G-RSV-Rev). For knockdown experiments, five pLKO1.puro lentiviral shRNA clones targeting *Hoatz* mRNA (TRCN0000190637, TRCN0000190708, TRCN0000201642, TRCN0000201968, TRCN0000201969) and a mock shRNA control (RHS4080) were obtained from Dharmacon (Lafayette, CO, USA). These lentiviral constructs were packaged into the VSV-G pseudotyped lentivirus and transduced into cultured mouse ependyma (Shamoto et al., 2018).

RNA *in situ* hybridization: *Hoatz*-specific digoxigenin (DIG)-labeled sense and antisense RNA probes were synthesized using a DIG RNA labeling kit (Roche Applied Science, Penzberg, Germany) and the linearized pGEM-T construct described above and then purified using an RNeasy Mini kit (Qiagen, Venlo, Netherlands). RNA *in situ* hybridization was performed as described (Kawaguchi et al., 2008).

Reverse transcription and polymerase chain reaction

Total RNA extraction, reverse transcription, and polymerase chain reaction were performed using Trizol reagent (Thermo Fisher Scientific), High Capacity cDNA reverse transcription kits (Thermo Fisher Scientific) and KOD Plus Neo DNA polymerase, respectively (Shamoto et al., 2018), following manufacture's instructions.

The same primer pair described above was used for semiquantitative analysis of *Hoatz* mRNA levels. The *Gapdh* primers were as follows: Gapdh Fwd, 5′-AACTTTGGCATTGTGGAAGG-3′; Gapdh Rev, 5′-ACACATTGGGGGTAGGAACA-3′. The annealing temperature for *Gapdh* was 60 °C. The concentrations of template cDNA samples were adjusted so that the amplicon of *Gapdh* at 25 cycles yielded similar band intensities on the agarose gel, determined using ImageJ (Schneider et al., 2012). PCR was performed for 27 cycles.

Real-time PCR analysis of *Eno4* mRNA: The Thunderbird SYBR qPCR mix (Toyobo) was used, and the amplicons were analyzed using a StepOnePlus Real-time PCR System (Thermo Fisher Scientific) following the manufacturer's instructions. The primer sequences were as follows: Eno4 Fwd, 5′-GGGTGAACGGATGACCAAATA-3′; Eno4 Rev, 5′-CCCCTCTGGATGAGTTCTTCCT-3′; B2m Fwd, 5′-CACTGACCGGCCTGTATGC-3′; B2m Rev, 5′-GGTGGCGTGAGTATACTTGAATTTG-3′. The annealing temperature was 59 °C.

Antibodies

Mouse anti-acetylated α-tubulin (6-11B-1) and rabbit anti-ENO4 (HPA037938) were procured from Sigma-Aldrich (St. Louis, MO). Rabbit anti-ARL13B (17711-1-AP) were obtained from Proteintech (Rosemont, IL). The anti-IFT88 antibody was provided by Prof. Gregory J Pazour, University of Massachusetts Medical School (Pazour et al., 2002). Mouse anti-pan actin (MS-1295-P0), rabbit anti-GFP (A-6455), and Alexa Fluor-conjugated secondary antibodies were from Thermo Fisher Scientific (Waltham, MA). Rabbit anti-DYKDDDDK as well as horseradish peroxidase-conjugated secondary antibodies were from Cell Signaling Technologies (Danvers, MA). Rabbit TrueBlot anti-rabbit IgG HRP (18-8816-33) was from Rockland Immunochemicals (Limerick, PA).

To generate rabbit polyclonal antibodies against HOATZ, the full-length mouse *Hoatz* cDNA, fused to a poly-histidine tag at its 3′ end and flanked by BamHI and XhoI restriction sites, was PCR-amplified and ligated to pGEX6P-1 (GE Healthcare Japan, Tokyo, Japan). The resulting construct was used to transform *Escherichia coli* strain BL21(DE3) (BioDynamics Laboratory, Tokyo, Japan). Recombinant GST-HOATZ-poly(His) was expressed, purified using His60 Ni Gravity Columns (Takara Bio, Shiga, Japan) in the presence of 6M guanidine hydrochloride, precipitated with 5% (v/v) trichloroacetic acid, and rinsed with acetone. The protein pellet (5.6 mg from 500 ml bacterial culture) was dissolved in 200 µl of the denaturing buffer and used to immunize rabbits at Genenet (Fukuoka, Japan). The IgG was purified using Protein G Sepharose 4 Fast Flow (GE Healthcare Japan). Affinity purification of anti-HOATZ antibodies was performed using a strip of PVDF membrane containing purified GST-HOATZ-poly(His) that was transferred from SDS-PAGE gel (Olmsted, 1981). The membrane was incubated with anti-HOATZ IgG diluted in TBST containing 10% bovine serum albumin and 2% sodium azide, and the bound antibodies were eluted with 0.1 M glycine buffer, pH 2.8. The eluate was neutralized immediately by 1M Tris-HCl, pH 8.0, concentrated with Vivaspin 500 column (Sartorius, Göttingen, Germany), supplemented with glycerol to a final concentration of 50% and stored at –30 °C.

Generation of Hoatz$^{-/-}$ mice using the CRISPR/Cas system

Hoatz was mutated through pronuclear injection of pX330 (Addgene plasmid #42230) according to Cong et al (Cong et al., 2013) and Mashiko et al (Mashiko et al., 2013), and in detail elaborated here. First, guide RNAs targeting exon 1 of mouse *Hoatz* (chromosome 9) were designed using the CRISPRdirect server program (Naito et al., 2014), and those targeting sequences immediately downstream of the first methionine codon with minimum off-targeting scores were selected. The following four oligonucleotide pairs were annealed and ligated into the BbsI site of pX330, to generate the clones designated T+05, T+08, T−03, and T−05: T+05 Fwd, 5′-caccAGGCTGTCCTAGCGGCAGGA-3′; T+05 Rev, 5′-aaacTCCTGCCGCTAGGACAGCCT-3′; T+08 Fwd, 5′-caccAATCTGCTCCCCTGGATTAC-3′; T+08 Rev, 5′-aaacGTAATCCAGGGGAGCAGATT-3′; T−03 Fwd, 5′-caccTGGGATTCCTTCCTGCCGCT-3′; T−03 Rev, 5′-aaacAGCGGCAGGAAGGAATCCCA-3′; T−05 Fwd, 5′-caccATCCAGGGGAGCAGATTTCC-3′; T−05 Rev, 5′-aaacGGAAATCTGCTCCCCTGGAT-3′. A 600-bp fragment of the mouse genome containing the target sites were cloned into the EcoRI-BamHI sites of pCAG EGxxFP (Addgene plasmid #50716) to generate the reporter plasmid for the *in vitro* double-strand break assay. The primer pairs were as follows: Fwd, 5′-gaattcAGATCTACGGAAGAAGAAACACAGGC-3′; Rev, 5′-ggatccGTGAGCCACCATGTGGTTGC-3′. The resulting pCAG EGxxFP and one of the pX330 constructs were used to cotransfect HEK293T cells using lipofection, and the efficiencies of the targeted double-strand break were determined by measuring green fluorescence using fluorescence microscopy and western blotting. Constructs of *Cetn1* (provided by Dr. Masahito Ikawa, Osaka University) (Mashiko et al., 2013) served as a control. Clone T−03, showing the highest double-strand break efficacy (supplemental Fig S1), was microinjected into BDF1×B6 zygotes at Advanced Biotechnology Center, University of Yamanashi. The resulting F0 animals were backcrossed with C57BL/6J, and the F1 progeny were genotyped to confirm germline transmission. The PCR primers used for genotyping of strain #3 were as follows: *Hoatz* exon1 Fwd, 5′-TCCACCGGTACTTCTCGGCCC-3′; *Hoatz* exon 1 Rev, 5′-CGCGCCACCGGTAGCCTCTG-3′. The annealing temperature was 65 °C.

Epididymal sperm were counted as previously described (Wang, 2003). Briefly, the cauda epididymis was isolated in PBS, weighed, and then cut into fine pieces in 1 ml of PBS using ophthalmic scissors and a stereomicroscope. After incubation at room temperature for 1 h, the sperm released into PBS were passed through a 100-µm mesh cell strainer to remove tissue debris, diluted as required, heat-inactivated, and counted using a hemocytometer.

Live imaging analyses

Analysis of the motility of ependymal cilia: FluoSpheres polystyrene microspheres (1.0-µm diameter, yellow-green fluorescence) (Thermo Fisher Scientific, Waltham, MA) were suspended in DMEM at approximately 1×10^8 beads/ml and added to a 35-mm glass-bottom culture dish (Nippon Genetics, Tokyo, Japan) containing a confluent primary culture of ependyma. After incubation for 3 min at room temperature, the cells were rinsed three times to remove excess beads and then observed using a fluorescence microscope. We previously found that the tips of the beating cilia occasionally adhered to the fluorescent microbeads. Thus, the beating of such “labeled”

cilia were traced using fluorescence microscopy (Katoh et al., 2018), and in detail elaborated here. At visible wavelengths, the adherent beads that beat at high frequencies appeared as short lines of fluorescence and were therefore distinguished from the other floating or nonbeating beads. We aimed to analyze the beating amplitude of motile cilia in cell culture using the “analyze particles” function of ImageJ. The images of the fluorescent microbeads on an ependymal monolayer were acquired at room temperature using an Olympus IX71 microscope equipped with UPlanSApo 10×/0.40 objective and DP72-cooled CCD color camera equipped with DP2-BSW software. The exposure time was 50-100 ms, and the image resolution was 1.54 pixels/μm.

The images capturing the traces of numerous beating beads were analyzed using ImageJ as follows: The acquired color images were first converted to 8-bit grayscale, and the background associated with a rolling ball radius of 20 was subtracted. A common threshold level that would best separate the bead tracings and background was then set for particle analysis. Then open “set measurement” dialog and select “shape descriptors” and “fit ellipse” options to measure the lengths of the major and minor axes of an ellipse fitting a selected region of interest and used to calculate the aspect ratio (major axis divided by minor axis). Using the same dialog, “Integrated density” was selected. Upon executing the “analyze particles” command, only 50–200 pixel^2 particles, circularities ranging from 0–0.80, were selected in the dialog window to exclude overlapped, aggregated, or static beads. Further, select “outlines” from the “show” option in the same dialog was used to determine if the regions of interest were properly selected and subsequently analyzed.

When a trace of a beating bead is analyzed, the values of the major and minor axes should correspond to the apparent beating amplitude and apparent bead diameter, respectively (Fig. 3B). The actual bead diameter was uniform and constant, although the apparent bead diameter measured using fluorescence microscopy varied depending on the degree of defocus. To minimize the influence of defocus on the measurement of the beating amplitude, the apparent beating amplitude was divided by the apparent bead diameter to calculate the normalized beating amplitude, which represents the aspect ratio of the normalized beating amplitude. Finally, a cumulative frequency distribution plot of the normalized beating amplitude was generated using GraphPad Prism software.

Analysis of the motility of sperm flagella: A pair of cauda epididyma was isolated, cut into small pieces using microscissors in 1-ml Leibovitz L-15 medium, and incubated at room temperature for 1 h. The samples were then filtered through 100-μm Falcon Cell Strainers (Corning, Corning, NY) to remove tissue debris, diluted (typically 1:2000), transferred to a 35 mm glass-bottom culture dish, and observed using an Olympus IX70 inverted microscope equipped with a 100-W mercury lamp, differential interference contrast optics, a UPlanSApo 20×/0.75 objective, and an Allied GE680 CCD camera. The images were recorded at 175 frames/s and analyzed using TI Workbench software (Fukatsu et al., 2004). The (number of frames per second)/(average number of frames of a single beat) (CBF) was calculated (Chilvers and O’Callaghan, 2000). To characterize flagellar beating patterns, we superimposed 10 representative frames acquired during one beating cycle.

Electron microscopy

Samples for SEM and TEM were fixed in half Karnovsky fixative (0.1 M cacodylate buffer, pH 7.4, 2 % paraformaldehyde, 2.5% glutaraldehyde) containing 0.2% tannic acid (Fujifilm Wako Pure Chemical, Osaka, Japan), then washed with 0.1 M cacodylate buffer containing 10% sucrose, post-fixed with 1% osmium tetroxide for 1 h on ice, and dehydrated with graded concentrations of ethanol (Narita et al., 2010, 2012; Nonami et al., 2013). For SEM analysis of sperm, samples suspended in ethanol were dried on a glass coverslip glued on a metal specimen mount, and sputter-coated with platinum. For TEM analysis, samples were further dehydrated with propylene oxide and embedded in epoxy resin.

Transverse sections of cilia from wild type and *Hoatz*$^{-/-}$ mouse trachea were analyzed as described elsewhere (Shoemark et al., 2012, 2020) to assess the ultrastructural integrity in a quantitative manner. Briefly, ultrathin sections of epon-embedded tissue specimens were stained with uranyl acetate and lead citrate and observed under JEM-2100F field emission electron microscope (JEOL, Tokyo, Japan) at an acceleration voltage of 120 kV. Digital images were acquired at 30,000–40,000 × magnifications using a bottom mount TemCam-F216 CMOS camera (TVIPS, Gauting, Germany). Then the 9+2 microtubules of >300 cilia and the dynein arms of >100 cilia were assessed to calculate the scores for each specimen, based on a definition of the hallmark diagnostic defects (class 1 defects) described in the international consensus guideline for reporting transmission electron microscopy results in the diagnosis of primary ciliary dyskinesia (Shoemark et al., 2020).

Sucrose density gradient centrifugation, immunoprecipitation, and shotgun proteomics

For sucrose density gradient fractionation, one pair of testes was isolated from an adult mouse and placed in ice-cold PBS. After measuring the wet weight (mg), the tunica albuginea was removed. The tissue sample was then transferred into a 2-ml glass/Teflon tissue grinder containing 1 ml of ice-cold LAP150 buffer (50 mM HEPES, pH 7.4, 150 mM KCl, 1 mM EGTA, 1 mM $MgCl_2$) containing 0.3% (v/v) Igepal CA-630, 1 mM dithiothreitol, and protease inhibitor cocktail (#P2714, Sigma-Aldrich) and was then homogenized (Nachury et al., 2007). After centrifugation at 10,000 × g for 10 min at 4 °C to remove debris, the supernatant was loaded onto a 9-ml 5%–40% linear sucrose gradient in LAP150 buffer containing 0.3% Igepal CA-630 and then centrifuged at 4 °C in a CP80WX Himac Utracentrifuge (Hitachi Koki, Tokyo, Japan) equipped with a Beckman SW41 rotor. The cumulative centrifugal effect ($\omega^2 t$) was set to 8.3×10^{11} rad^2/s. The sedimentation standards bovine pancreas chymotrypsinogen A (2.6S), bovine serum albumin (4.6S), bovine liver catalase (11.3S), and horse spleen apoferritin (17.6S) were from Sigma-Aldrich. After centrifugation, 13 fractions (approximately 770 µl each) were collected from the top of the tube using a DGF-U fractionator (Hitachi Koki, Tokyo, Japan).

For immunodetection, the proteins in the density gradient fractions were precipitated with 5% (v/v) trichloroacetic acid, rinsed twice with cold methanol, dried briefly, and dissolved in 4× SDS sample buffer. To adjust the loading between different animals, the sample buffer was added to each fraction tube at 50 µl per 60 mg of tissue weight measured before homogenization.

For immunoprecipitation and shotgun proteomics, the fractions #3–#8 containing HOATZ were pooled, and an aliquot (approximately 1.5 ml) was incubated for 3 h at 4 °C with 50 μl of Dynabeads Protein G (Veritas, Tokyo, Japan) that was crosslinked to affinity-purified anti-HOATZ antibodies. The beads were then washed three times, 10 min each, with LAP150 buffer containing 0.3% Igepal CA-630; rinsed twice briefly with the same buffer without detergent; and incubated for 2 min in 30 μl of 0.1 M glycine buffer, pH 2.8, to elute the bound proteins. The eluate was transferred to a new tube and neutralized with 3 μl of 1 M Tris-HCl, pH 8.0. These steps were repeated twice, with the final incubation overnight instead of 3 h. The eluates were pooled, digested with trypsin and subjected to shotgun proteomic analyses using Dina-2A nanoflow LC system (KYA Technologies, Tokyo, Japan) coupled with LTQ-Orbitrap Velos mass spectrometer (Thermo Fisher Scientific, Bremen, Germany) (Contu et al., 2017). The samples were injected into a 75 μm reversed-phase C18 column at a flow rate of 10 μl/min, and eluted with a linear gradient of solvent A (2% acetonitrile and 0.1% formic acid in H_2O) to solvent B (40% acetonitrile and 0.1% formic acid in H_2O) at 300 nl/min. The separated peptides were then sequentially sprayed from a nanoelectrospray ion source (KYA Technologies) and analyzed by collision-induced dissociation. The analyses were operated in data-dependent mode, switching automatically between MS and tandem mass spectrometry (MS/MS) acquisition. For collision-induced dissociation analyses, full-scan MS spectra (from 380 to 2000 *m/z*) were acquired in the orbitrap with resolution of 100,000 at 400 *m/z* after ion count accumulation to the target value of 1,000,000. The 20 most intense ions at a threshold above 2000 were fragmented in the linear ion trap with normalized collision energy of 35% for an activation time of 10 ms. The orbitrap analyzer was operated with the 'lock mass' option to perform shotgun detection with high accuracy. Protein identification was conducted by searching MS and MS/MS data against the RefSeq (National Center for Biotechnology Information) mouse protein database (29,579 protein sequences as of 4 February, 2013) by Mascot version 2.5.1 (Matrix Science). Methionine oxidation, protein N-terminal acetylation, and pyroglutamination for N-terminal glutamine, were set as variable modifications. A maximum of two missed cleavages was allowed in our database search, while the mass tolerance was set to 3 ppm for peptide masses and 0.8 Da for MS/MS peaks, respectively. In the process of peptide identification, we conducted decoy database searching by Mascot and applied a filter to satisfy a false positive rate <1%.

Western blotting and immunohistochemistry

Western blotting and immunohistochemistry were performed following standard protocols (Narita et al., 2015). In brief, for western blotting, proteins resolved by SDS-PAGE were transferred onto PVDF membrane using a wet transfer system, at 5V overnight at room temperature. The resulting blots were blocked with 10% skim milk in TBST, incubated with the primary antibodies overnight at 4 °C, washed three times with TBST, incubated with HRP-conjugated secondary antibodies for 1 hour at room temperature, washed three times again, and incubated with Chemi-Lumi One Super substrate (Nakalai Tesque, Kyoto, Japan) in ImageQuant LAS-4000 lumino analyzer (GE Healthcare Japan) for detection. For immunohistochemistry, sections of 4% paraformaldehyde-fixed tissues on glass slides were autoclaved in 20 mM Tris-HCl, pH 9.0, containing 10% sucrose for heat-induced

epitope retrieval, and blocked with 10% skim milk in TBST. Then the samples were incubated with the primary antibodies in the blocking buffer overnight at 4 °C, washed three times with TBST, incubated with the Alexa-conjugated secondary antibodies for 2 hours at RT, washed again, and sealed with an antifade mounting medium containing DAPI. For the immunohistochemical analysis of testis sections for ENO4 expression, the tissue was cut in half by razor blades, fixed with 4% paraformaldehyde in PBS by immersion, embedded in OCT compound, and cut into 5 μm thick slices onto a glass slide. The sections were dried, boiled for 5 min in 10 mM citrate buffer, pH 6.0, for epitope retrieval, washed three times in PBS containing 0.1% Triton X-100, and blocked with 20% goat serum in PBST (Dong et al., 2014).

For western blotting, primary antibodies were diluted 1:500, except anti-AcTub (1:1000). Antibody dilutions used for immunohistochemistry were as follows: acetylated α-tubulin (1:1,000), ARL13B (1:500), and ENO4 (1:50).

Statistical analysis

The significance of differences between two groups were evaluated using two-tailed *t* tests. Differences between multiple groups were analyzed using the nonparametric Kruskal–Wallis test that does not assume a Gaussian distribution, followed by Dunn's multiple comparison post hoc test. $P < 0.05$ indicates a significant difference.
